# Overexpression of Medicago *MtCDFd1_1* Causes Delayed Flowering in Medicago *via* Repression of *MtFTa1* but Not *MtCO*-Like Genes

**DOI:** 10.3389/fpls.2019.01148

**Published:** 2019-09-19

**Authors:** Lulu Zhang, Andrew Jiang, Geoffrey Thomson, Megan Kerr-Phillips, Chau Phan, Thorben Krueger, Mauren Jaudal, Jiangqi Wen, Kirankumar S. Mysore, Joanna Putterill

**Affiliations:** ^1^The Flowering Lab, School of Biological Sciences, University of Auckland, Auckland, New Zealand; ^2^Noble Research Institute, Ardmore, OK, United States

**Keywords:** *CYCLING DOF FACTOR*, *MtCDFd1_1*, *MtFTa1*, *MtFTb*, *CO*, *Medicago*, flowering time, primary axis elongation

## Abstract

Optimizing flowering time is crucial for maximizing crop productivity, but gaps remain in the knowledge of the mechanisms underpinning temperate legume flowering. Medicago, like winter annual Arabidopsis, accelerates flowering after exposure to extended cold (vernalization, V) followed by long-day (LD) photoperiods. In Arabidopsis, photoperiodic flowering is triggered through CO, a photoperiodic switch that directly activates the FT gene encoding a mobile florigen and potent activator of flowering. In Arabidopsis, several CYCLING DOF FACTORs (CDFs), including AtCDF1, act redundantly to repress CO and thus FT expression, until their removal in LD by a blue-light-induced F-BOX1/GIGANTEA (FKF1/GI) complex. Medicago possesses a homolog of FT, MtFTa1, which acts as a strong activator of flowering. However, the regulation of MtFTa1 does not appear to involve a CO-like gene. Nevertheless, work in pea suggests that CDFs may still regulate flowering time in temperate legumes. Here, we analyze the function of Medicago MtCDF genes with a focus on MtCDFd1_1 in flowering time and development. MtCDFd1_1 causes strong delays to flowering when overexpressed in Arabidopsis and shows a cyclical diurnal expression in Medicago with peak expression at dawn, consistent with AtCDF genes like AtCDF1. However, MtCDFd1_1 lacks predicted GI or FKF1 binding domains, indicating possible differences in its regulation from AtCDF1. In Arabidopsis, CDFs act in a redundant manner, and the same is likely true of temperate legumes as no flowering time phenotypes were observed when MtCDFd1_1 or other MtCDFs were knocked out in Medicago Tnt1 lines. Nevertheless, overexpression of MtCDFd1_1 in Medicago plants resulted in late flowering relative to wild type in inductive vernalized long-day (VLD) conditions, but not in vernalized short days (VSDs), rendering them day neutral. Expression of MtCO-like genes was not affected in the transgenic lines, but LD-induced genes MtFTa1, MtFTb1, MtFTb2, and MtSOC1a showed reduced expression. Plants carrying both the Mtfta1 mutation and 35S:MtCDFd1_1 flowered no later than the Mtfta1 plants. This indicates that 35S:MtCDFd1_1 likely influences flowering in VLD via repressive effects on MtFTa1 expression. Overall, our study implicates MtCDF genes in photoperiodic regulation in Medicago by working redundantly to repress FT-like genes, particularly MtFTa1, but in a CO-independent manner, indicating differences from the Arabidopsis model.

## Introduction

Plants integrate several molecular pathways to control when they flower to maximize reproductive fitness and successful development of seeds and fruit (Fornara et al., 2010; Srikanth and Schmid, 2011; Andrés and Coupland, 2012). One of these pathways involves the responsiveness to changes in day length (photoperiod), which plays a vital role in the plant’s ability to synchronize flowering time with favorable seasonal conditions (Putterill et al., 2004). For example, in temperate plants such as winter annual *Arabidopsis thaliana* (*Arabidopsis*) and the legume *Medicago truncatula* (*Medicago*), extended winter cold (vernalization, V) followed by exposure to long-day (LD) photoperiods—a feature of spring and early summer—promotes flowering.

The well-characterized *Arabidopsis* LD pathway promotes flowering *via* the accumulation of CONSTANS (CO) protein in the leaves, which directly activates the expression of the potent floral activator *FLOWERING LOCUS T* (*FT*) in the late afternoon of LD, but not in short days (SDs). *FT* encodes a mobile florigen that moves to the shoot apical meristem and initiates the transition to flowering *via* activation of genes such as *SUPPRESSOR OF OVEREXPRESSION OF CONSTANS 1* (*SOC1*; Andrés and Coupland, 2012). Several factors converge to facilitate the accumulation of CO protein in LD including releasing the *CO* gene from transcriptional repression by CYCLING DOF FACTOR (CDF) transcription factors. This occurs *via* the light-induced formation of the FLAVIN-BINDING, KELCH REPEAT, F-BOX1/GIGANTEA (FKF1/GI) complex which targets the CDFs for degradation *via* the proteasome, which in turn enables the transcription of *CO* (Imaizumi et al., 2005; Sawa et al., 2007; Fornara et al., 2009; Song et al., 2012; Goralogia et al., 2017). In addition, there is direct regulation of *FT* by AtCDF1 (Song et al., 2012).

The acceleration of flowering by *FT-like* genes is conserved in a diverse range of species (Wickland and Hanzawa, 2015; Putterill and Varkonyi-Gasic, 2016) including the *FTa1* gene in the temperate legumes *Pisum sativum* (pea) and *Medicago* (Hecht et al., 2011; Laurie et al., 2011). Temperate legumes are of particular interest as many serve as important agricultural crops with flowering time playing a significant role in annual production yields (Graham and Vance, 2003; Weller and Ortega, 2015).

However, increasing evidence suggests that temperate legume species operate with a *CO*-independent mechanism for the regulation of *FT-like* genes and thus flowering (Putterill et al., 2013; Weller and Ortega, 2015). Analysis of *Medicago CO-like* (*COL*) genes revealed that they were unable to complement the *Arabidopsis co* null mutant and did not promote flowering when overexpressed (Wong et al., 2014). *Medicago col* null mutant lines did not have a flowering phenotype under LD and therefore were unlikely to be involved in the *Medicago* photoperiodic response (Wong et al., 2014). An additional difference is that there are three LD-induced *FT* genes in *Medicago*, but none have the same diurnal pattern of expression as *Arabidopsis FT*, suggesting a different regulatory mechanism (Laurie et al., 2011). Thus, there is a substantial knowledge gap in our understanding of photoperiodic flowering in these species (Hecht et al., 2005, Hecht et al., 2011; Laurie et al., 2011; Putterill et al., 2013; Weller and Ortega, 2015; Ridge et al., 2016).

Despite the apparent lack of a functional *CO* in temperate legumes, legume *CDF*s appear to still participate in photoperiodic flowering. Specifically in garden pea, the dominant late-flowering *LATE2* mutant was recently mapped to a *CDF* homolog, *PsCDFc1*. Yeast two-hybrid assays indicate that the mutation disrupts the binding of PsFKF1 to PsCDFc1, indicating that increased PsCDFc1 protein stability may be the basis of the dominant phenotype (Ridge et al., 2016). Plants carrying the *late2/Pscdfc1* mutation have reduced expression of LD-induced *FT-like* genes, but not *PsCOL* genes. This indicates that CDFs participate in the photoperiodic regulation of flowering in pea but that the mechanism differs to that of *Arabidopsis* (Ridge et al., 2016).

*CDFs* were first characterized in *Arabidopsis* and are a subset of the plant-specific DNA-binding One Zinc Finger (DOF) gene family of transcription factors (Yanagisawa, 2002; Noguero et al., 2013). They are distinguished by their cyclical diurnal transcript levels, with the majority of genes showing peak transcript accumulation early in the day. In *Arabidopsis*, CDFs have an overlapping role in photoperiodic flowering control as single *AtCDF* mutants have either no or only weak flowering time phenotypes, but a quadruple *Atcdf1–3,5* mutant has day-neutral early flowering (Imaizumi et al., 2005; Fornara et al., 2009).

In *Medicago*, phylogenetic analysis has revealed a total of 42 *Medicago* DOF proteins clustered into four phylogenetic clades (Shu et al., 2015). One of these clades, MCOGD, contains all of the 13 MtCDF-like proteins, which in turn group into several subclades (Shu et al., 2015; Ridge et al., 2016). These are expressed predominantly in leaf blades, nodules, and buds (Shu et al., 2015), with expression in leaves consistent with a role in photoperiodic flowering (Turck et al., 2008).

Here, we analyze the function of *MtCDF* genes in the regulation of *Medicago* flowering, with a focus on *MtCDFd1_1*. We analyzed the gene expression patterns of *MtCDF*s in VLD and VSD RNA-Seq morning time courses and surveyed plants carrying transposon insertions in *MtCDF* genes. While flowering time phenotypes were not observed in individual *Medicago* mutants, overexpressing the genes in *Arabidopsis* identified five genes, including *MtCDFd1_1*, which caused strong delays to flowering. We then examined the effect of overexpressing *MtCDFd1_1* in *Medicago* on plant development, flowering time, and the expression of known flowering time genes. Collectively, our results implicate *MtCDF* genes as regulators of photoperiodic flowering and plant architecture *via* the repression of *FT-like* genes, such as *MtFTa1*.

## Materials And Methods

### Bioinformatics

Legume and other plant CDF protein sequences were obtained from the literature (Shu et al., 2015; Ridge et al., 2016) and by BLASTP searches with AtCDF1 of the J. Craig Venter Institute (JCVI) *Medicago* genome (Mt4.0 http://www.jcvi.org/medicago/) and National Center for Biotechnology Information (NCBI) (http://www.ncbi.nlm.nih.gov/). The *Medicago MtCDF* gene identifiers and names are listed in **Table S1**. The phylogenetic tree of CDF-like proteins from *Medicago*, other legumes, tomato, potato, and *Arabidopsis* was constructed by aligning full-length amino acid sequences using MUSCLE (version 3.8.425; Edgar, 2004) as implemented in (version 11.1.5) and using the neighbor-joining algorithm implemented in PAUP* (version 4.0; Swofford, 2003). An existing RNA-Seq dataset (Thomson et al., 2019) comprising three biological replicates was consulted to obtain the mean abundance of *MtCDF-like* gene transcripts in leaf tissue at 0, 2, and 4 h after dawn in transcripts per million (TPMs) in SD and LD photoperiods. *Medicago Tnt1* retroelement insertion lines were identified by screening the FST database (https://medicago-mutant.noble.org/mutant/blast/blast.php) and are listed in **Table S1**.

### Plant Materials and Growth Conditions

*Medicago truncatula* (*Medicago*) wild type Jester (Hill, 2000) and R1 08-1_C3 (R108; Trinh et al., 1998) used in this study belong to *Medicago truncatula* Gaertn (barrel medic), ssp. *truncatula* and ssp. *tricycla*, respectively. All *Tnt1* insertion mutants in the R108 background listed in **Table S1** were obtained from the Noble Research Institute, LLC (Ardmore, OK, USA). *Arabidopsis thaliana* (*Arabidopsis*) wild type Columbia was used. The *Mtfta1* mutant utilized was NF1634 (Jaudal et al., 2019).

*Medicago* and *Arabidopsis* plants were grown in controlled environments under ∼200 μM m^−2^ s^−1^ cool white fluorescent light at 22°C or 24°C and under ∼140 μM m^−2^ s^−1^ at 22°C, respectively, in LDs (16 h light/8 h dark) or SDs (8 h light/16 h dark), with or without prior vernalization of germinated seeds at 4°C for 21 days, as previously described (Laurie et al., 2011; Yeoh et al., 2013; Jaudal et al., 2015). *Medicago* flowering time was measured in days to when the first floral bud was observed by eye and the number of nodes on the primary axis at flowering. *Arabidopsis* flowering time was measured in days to when the first floral buds were observed by eye and the total number of rosette and cauline leaves at flowering.

*CaMV 35S* overexpression constructs were made by inserting complementary DNA (cDNA) sequences into vector pB2GW7 (Karimi et al., 2007) using Gateway^®^ Technology (Invitrogen^®^, CA, USA). Forward and reverse primers used for Gateway cloning are shown in **Table S2**. Transgenic *Arabidopsis* plants overexpressing *MtCDF* genes were generated using *Agrobacterium tumefaciens* GV3101 containing overexpression constructs *via* floral dipping and Basta selection of the T_1_ population as previously described (Martinez-Trujillo et al., 2004; Jaudal et al., 2015).

Transgenic R108 *Medicago* plants overexpressing *MtCDFd1_1* were generated using *A. tumefaciens* EHA105 with the *35S:MtCDFd1_1* construct *via* somatic embryogenesis and subsequent BASTA selection in soil as previously described (Cosson et al., 2006; Laurie et al., 2011).

*35S:MtCDFd1_1* plants and *Mtfta1* heterozygous plants were crossed together (Chabaud et al., 2006) and then bred and genotyped to identify F_2_ *35S:MtCDFd1_1/Mtfta1* homozygous mutant plants.

### Quantitative Reverse Transcriptase PCR (qRT-PCR) for Gene Expression

RNA extraction and cDNA synthesis using an oligo dT primer was carried out as previously described (Laurie et al., 2011; Yeoh et al., 2013; Jaudal et al., 2015). qRT-PCR was performed using SYBR^®^ green chemistry on Applied Biosystems^®^ 7900HT Sequence Detection System (Applied Biosystems^®^, CA, USA) or QuantStudio™ 5 Real-Time PCR System (Applied Biosystems^®^, CA, USA). Each data point is derived from three biological replicates harvested in parallel. Each replicate consisted of a pool of leaf tissue from either two or three independent plants. Primer sequences used for qRT-PCR are listed in **Table S2**. Gene expression was calculated using the comparative Ct method (Livak and Schmittgen, 2001) with modifications (Bookout and Mangelsdorf, 2003). Samples were normalized to *PROTEIN PHOSPHATASE 2A* (*PP2A*; *Medtr6g084690*).

The statistical testing for the gene expression data presented in **Figures 4** and **6** was performed using a one-way analysis of variance (ANOVA) test between the means (α = 0.05). The Shapiro–Wilk normality assumption test was performed on all data presented. Multiple pairwise comparisons adjusted for false discovery rate (FDR) were utilized to highlight statistically significant differences in the data presented.

### Yeast Two-Hybrid Assays

Full-length coding sequences of *MtCDFd1_1*, *MtCDFc1*, and *AtCDF1* and the KELCH-repeat region of *AtFKF1* (amino acids 284 to 619; Imaizumi et al., 2005; Ridge et al., 2016) were used for the yeast two-hybrid assay. Gene fragments were cloned into Invitrogen destination vectors pDEST22 (AD, prey) and pDEST32 (BD, bait). The prey and bait constructs were transformed into the haploid yeast strains PJ69-4A and PJ69-4α (James et al., 1996), respectively, and selected on synthetic defined (SD) medium lacking tryptophan (Trp; prey) or leucine (Leu; bait). PJ69-4A and PJ69-4α strains were then mated, and diploid clones with both constructs were selected on medium lacking Trp and Leu (SD −Trp −Leu). Haploids containing empty pDEST22 and pDEST32 were also included to test autoactivation. Two independent diploid clones from each mating were diluted in 100 µl of water and plated on nonselective medium (SD −Trp −Leu) and selective medium [SD −Trp −Leu −histidine (His)] with different 3-amino-1,2,4-triazol (3-AT) concentrations (0, 1, 2, 10, 25, 50, and 100 mM). Colonies developed over 11 days at 28°C. Photos were taken on days 4, 7, 9, and 11. Similar results were obtained for each of the two independent clones. The positive control interactors were AtCDF1 and AtFKF1.

## Results

### Initial Characterization of 14 *MtCDF* Genes

**Figure 1:**
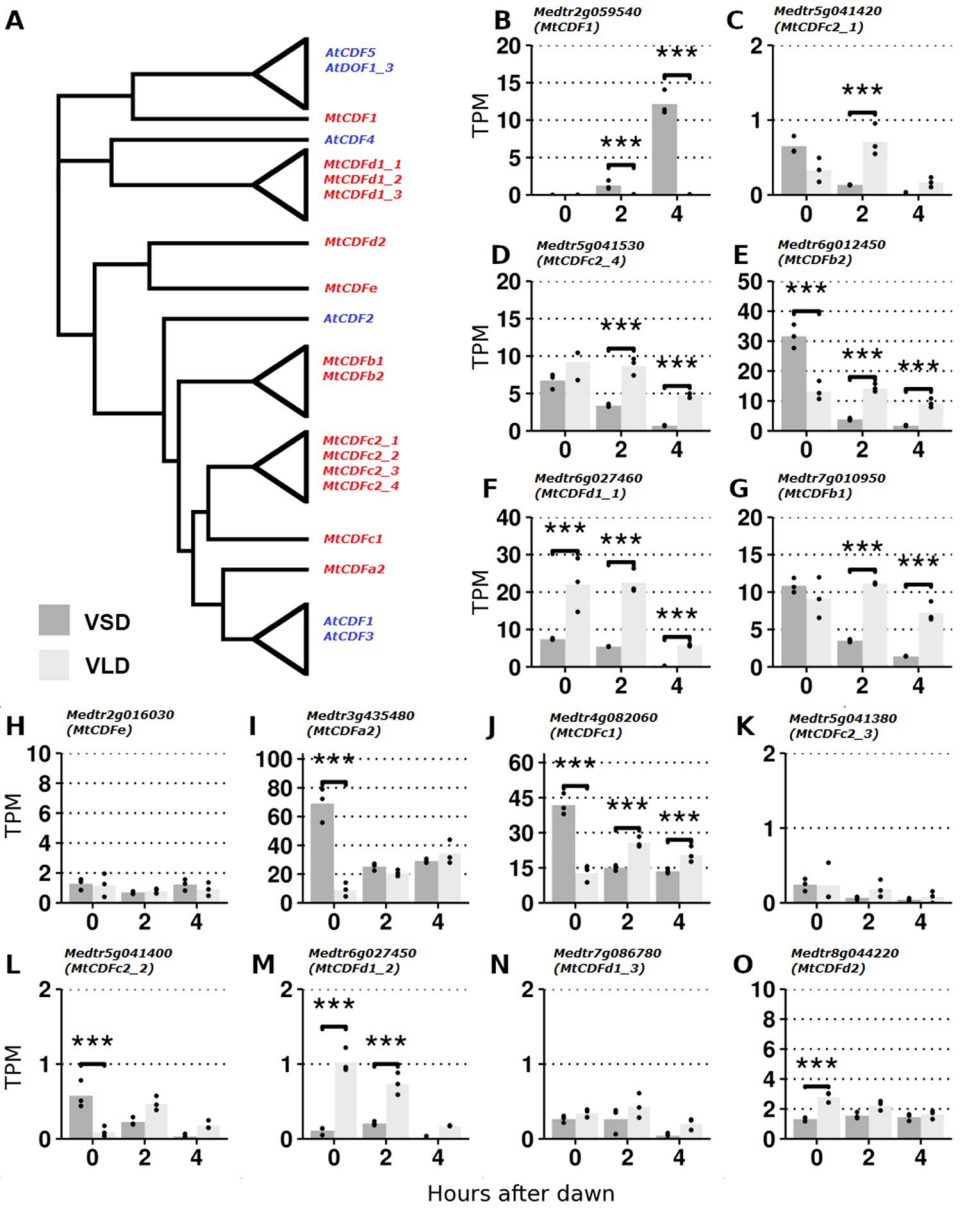
RNA-Seq analysis of *MtCDF* gene expression in *Medicago* Jester leaf tissue under vernalized short-day (VSD) and vernalized long-day (VLD) photoperiods. **(A)** Neighbor joining tree diagram of CDF-like proteins in *Arabidopsis* and *Medicago* using their full-length amino acid sequences. Clades of similar proteins were collapsed. See **Figure S1** for a more comprehensive tree. **(B**–**O)** Derived from RNA-Seq data (Thomson et al., 2019); the mean abundance of *MtCDF* gene transcripts in leaf tissue at 0, 2, and 4 h after dawn in transcripts per million (TPMs) in VSD and VLD. Abundances for the three biological replicates are plotted as points, and asterisks indicate significant differential expression (Wald significance tests; α = 0.05).

To investigate the role of *MtCDF* genes in *Medicago* flowering time, we selected 14 *MtCDF* genes for initial analysis. These were 13 *MtCDF*s identified previously (Shu et al., 2015; Ridge et al., 2016) and a 14th related gene (*MtCDF1*) that we previously observed to have cyclical diurnal expression with an afternoon peak (Thomson et al., 2019). **Table S1** lists the *MtCDF* gene identifiers (JCVI Medicago genome Mt4.0) and corresponding gene names following the nomenclature in Ridge et al. (2016). The phylogenetic groupings of the predicted proteins along with AtCDFs are shown in **Figure 1A**, with a more comprehensive phylogenetic tree containing additional CDF proteins from legumes, tomato, and potato shown in **Figure S1**.

Protein sequence alignments of *Medicago* and *Arabidopsis* CDFs (**Figure S2**) highlighted the highly conserved DOF domain in all the MtCDF proteins and MtCDF1. However, five proteins (MtCDF1, MtCDFd1_1, MtCDFd1_2, MtCDFd1_3, and MtCDFe) and two *Arabidopsis* CDFs (AtCOG1 and AtCDF4) lacked the two C-terminal regions that in *Arabidopsis* function as FKF1- and GI-binding domains. Two MtCDFs (MtCDF1 and MtCDFe) also lacked the predicted N-terminal TOPLESS (TPL)-binding domain. Recently, CDFs in *Arabidopsis* have been shown to form a complex with TPL (Goralogia et al., 2017); hence, the lack of TPL domains in these MtCDFs may indicate a functional divergence.

We analyzed expression of the 14 *MtCDF* genes (**Figure 1**) in an RNA-Seq dataset (Thomson et al., 2019) derived from leaves of plants grown in LD and SD after vernalization (V) and harvested at three time points: dawn and 2 and 4 h after dawn. We detected reads mapping to all 14 *MtCDF* genes, confirming that they are expressed in leaves as previously observed (Shu et al., 2015) and consistent with a potential role in photoperiodic flowering.

Transcript abundance varied >70-fold between the genes (**Figures 1B–O**). The four most abundant were *MtCDFa2*, *MtCDFc1*, *MtCDFb2*, and *MtCDFd1_1*. Most genes (11/14; *MtCDF1*, *MtCDFa2*, *MtCDFb1*, *MtCDFb2*, *MtCDFc1*, *MtCDFc2_1*, *MtCDFc2_2*, *MtCDFc2_4*, *MtCDFd1_1*, *MtCDFd1_2*, and *MtCDFd2*) were significantly differentially expressed between the two photoperiods. These included three genes, *MtCDFd1_1*, *MtCDFb2*, and *MtCDFc1*, that were differentially expressed between the photoperiods at all three time points.

Further analysis of *MtCDFd1_1* by qRT-PCR over a full day (**Figure S3**), indicated that the transcript of this gene has a diurnal cycle that is modulated by LD and SD photoperiods similar to the *Arabidopsis CDFs* (*AtCDF1-3,5*; Imaizumi et al., 2005; Fornara et al., 2009).

### No Altered Flowering Time Phenotypes Were Observed in Medicago *MtCDF Tnt1* Insertion Lines

To investigate the function of the *MtCDF* genes, we screened the *Medicago Tnt1* flanking sequence database for candidate mutant *Medicago* plant lines with knockout *Tnt1* retroelement insertions in *MtCDF* genes. The results are summarized in **Table S1**. Lines homozygous for *Tnt1* insertions in 13 out of the 14 genes (the exception was *MtCDFd2*) were found.

In total, we identified 27 candidate plant lines, genotyped them for the presence of the *Tnt1* insertion, examined their gene expression, and scored their flowering time in VLD, LD, and VSD. Knockout, or knockdown, of gene expression was confirmed by qRT-PCR in 11/13 homozygous lines, except *MtCDFb1* and *MtCDFb2*, where the insertions were located in introns. However, no altered flowering time phenotypes were observed in any single mutant, which may be attributable to functional redundancy between some of the genes, as observed in *Arabidopsis* (Fornara et al., 2009).

### Overexpression of *MtCDFd1_1* and Four Other *MtCDF* Genes Causes Delayed Flowering in Arabidopsis

In previous work, overexpression of *AtCDF* genes, including *AtCDF1*, caused delayed *Arabidopsis* flowering (Imaizumi et al., 2005; Fornara et al., 2009). On the other hand, overexpression of wild-type pea *PsCDFc1* in *Arabidopsis* did not give late-flowering transgenic plants (Ridge et al., 2016). Only overexpression of the mutant version of *PsCDFc1* from the *late2* mutant resulted in late-flowering *Arabidopsis* plants (Ridge et al., 2016).

Here, having not observed mutant phenotypes in *Medicago MtCDF* knockout lines (**Table S1**), we turned to *Arabidopsis* to use as a rapid heterologous system for testing if any of the *MtCDFs* might regulate *Arabidopsis* flowering time. If such *MtCDF* genes were to be identified in this screen, then one would be selected for the overexpression functional analysis in *Medicago*.

**Figure 2:**
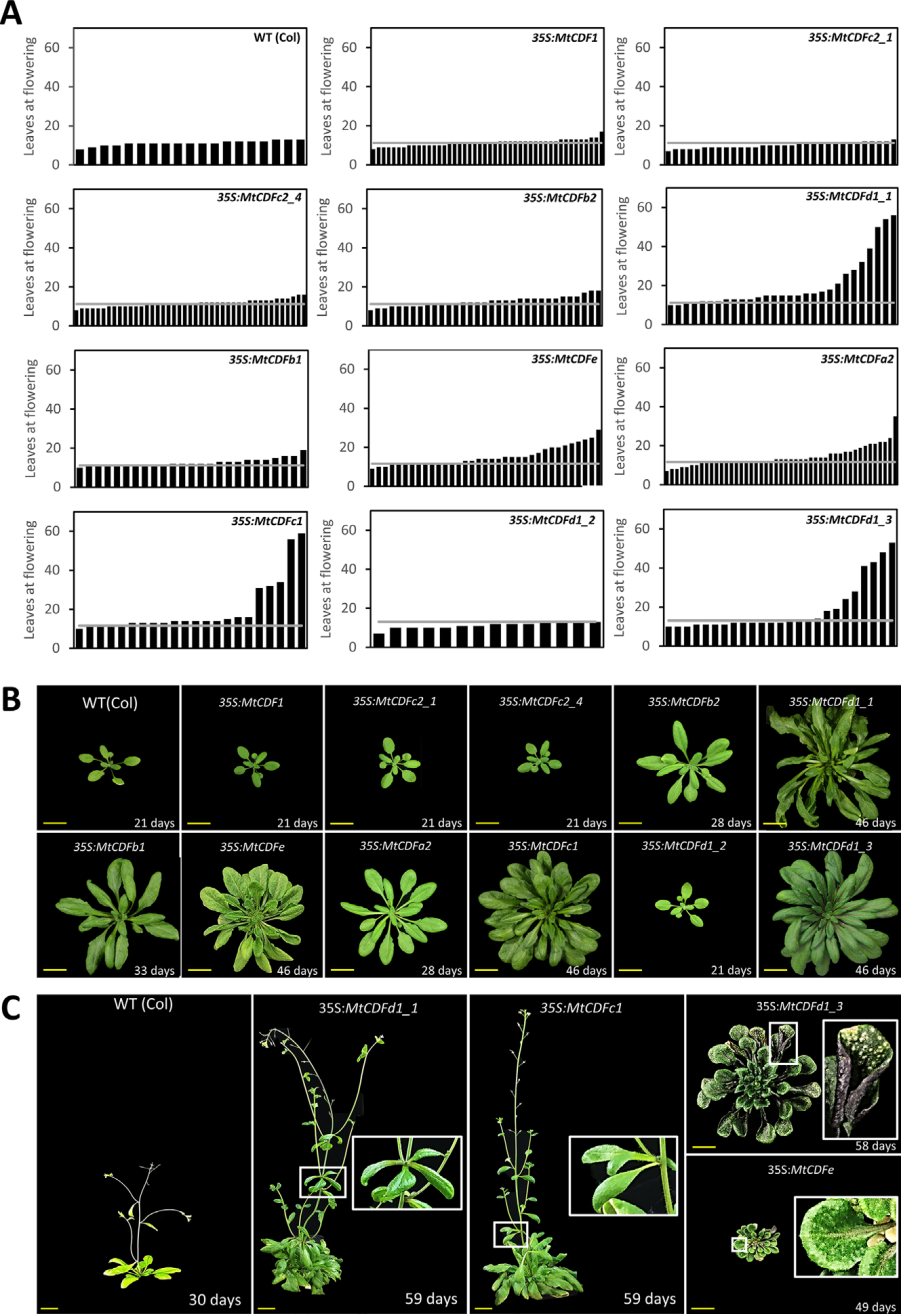
Overexpression of *Medicago CDF* genes in *Arabidopsis* can result in late flowering. **(A)** Flowering time of independent T_1_ transgenic plants (*n* ≥ 14) derived from 11 *35S:MtCDF* expression vectors and Columbia wild-type *Arabidopsis* in LD conditions. The gray line represents the average leaves at flowering for Columbia; 11.2 ± 0.63 leaves (t.SE 0.05; *n* = 19). **(B)** Photographs of selected T_2_- and T_3_-generation *35S:MtCDF* plants at the time of flowering. **(C)** Several *35S:MtCDFd1_1* and *35S:MtCDFc1* transgenic plants displayed aerial rosette phenotypes (white boxes) and poor fertility. Multiple *35S:MtCDFd1_3* and *35S:MtCDFe* transgenic plants had an upright rosette leaf stature with rigid long-handle spoon-shaped leaves. Additionally, these plants were darker in color with purple abaxial surfaces but had light-colored spots on the older leaves (white boxes in the last panel) and had poor fertility. Age of the plants indicated in days. Yellow scale bars = 2 cm.

We constitutively expressed 11 genes (*MtCDF1*, *MtCDFa2*, *MtCDFb1*, *MtCDFb2*, *MtCDFc1*, *MtCDFc2_1*, *MtCDFc2_4*, *MtCDFd1_1*, *MtCDFd1_2*, *MtCDFd1_3*, and *MtCDFe*) from across different subclades in wild-type *Arabidopsis* and measured flowering time (**Figure 1A** and **Figure S1**). Expression constructs were made by fusing the *MtCDF*s to the 35S promoter and then introduced into wild-type Columbia plants with the flowering time of T_1_ *Arabidopsis* transformants and photographs of selected T_2_ and T_3_ progeny presented in **Figure 2**.

Overexpression of five of the genes tested (*MtCDFa2*, *MtCDFc1*, *MtCDFd1_1*, *MtCDFd1_3*, and *MtCDFe*) resulted in strong delays to flowering in multiple independent T_1_ lines in LD, compared to Columbia (**Figures 2A, B**). Interestingly, these genes arise from different *MtCDF* subclades (**Figure 1A** and **Figure S1**). Overexpression of two other genes (*MtCDFb1* and *MtCDFb2*) produced several transgenic plants that showed a slight delay in flowering time, while overexpression of four genes (*MtCDF1*, *MtCDFc2_1*, *MtCDFc2_4*, and *MtCDFd1_2*) had little to no effect on Columbia flowering time (**Figures 2A, B**).

Apart from being late flowering, unusual aerial architectural phenotypes were seen compared to *Arabidopsis* Columbia plants (**Figure 2C**). Specifically, an abnormal late-flowering phenotype characterized by aerial rosettes and poor fertility was observed in several independent transgenic lines carrying either of two transgenes, *35S:MtCDFd1_1* or *35S:MtCDFc1*. The aerial rosette phenotype is a feature also seen in some *Arabidopsis* plants where the floral transition is delayed including resulting from disruptions in the floral transition genes including *SOC1*, *AGAMOUS-like 42* (*AGL42*), *AGL71*, *AGL72* (Dorca-Fornell et al., 2011), *FT*, *TWIN SISTER OF FT* (*TSF*; Hiraoka et al., 2013), *FLOWERING LOCUS C* (*FLC*), *FRIGIDA* (*FRI*), and *AERIAL ROSETTE 1* (*ART1*; Poduska et al., 2003).

In addition, multiple independent lines carrying either of two transgenes, *35S:MtCDFe* or *35S:MtCDFd1_3*, displayed an upright rosette leaf stature with rigid, long-handled spoon-shaped leaves (**Figure 2C**). These plants also were smaller than wild type, infertile with a lack of primary inflorescence bolting, and darker in color. In addition, in some *35S:MtCDFd1_3* lines, the older leaves of some plants developed spotty lesions (**Figure 2C**).

In summary, among the *MtCDF*s, *MtCDFa2*, *MtCDFc1*, *MtCDFd1_1*, *MtCDFd1_3*, and *MtCDFe* were able to cause strong delays to flowering in multiple transgenic lines when overexpressed in wild-type *Arabidopsis*. The remaining *MtCDF* genes we tested did not appear to have much effect on flowering time in *Arabidopsis* in our experiments, but this may be due to factors such as transgene expression level.

### Constitutive Expression of *MtCDFd1_1* in Medicago Causes Late Flowering in VLD

We selected *MtCDFd1_1* for further functional analysis by overexpression in *Medicago*. This was because its transcript was relatively abundant in *Medicago* leaves and exhibited diurnal cycling in VLD and VSD similar to *AtCDF*s that regulate flowering time redundantly in *Arabidopsis*. Additionally, it caused a strong delay to flowering in multiple independent lines when overexpressed in *Arabidopsis*. However, it was interesting also because its predicted protein sequence differs from these AtCDF proteins and from PsCDFc1/LATE2, which has already been characterized in pea (Ridge et al., 2016), falling into a different subclade (d1, **Figure S1**). It lacks the predicted GI- and FKF1-binding domains (**Figure S2**) and appears not to interact with AtFKF1 in yeast two-hybrid assays (**Figure S4**).

**Figure 3:**
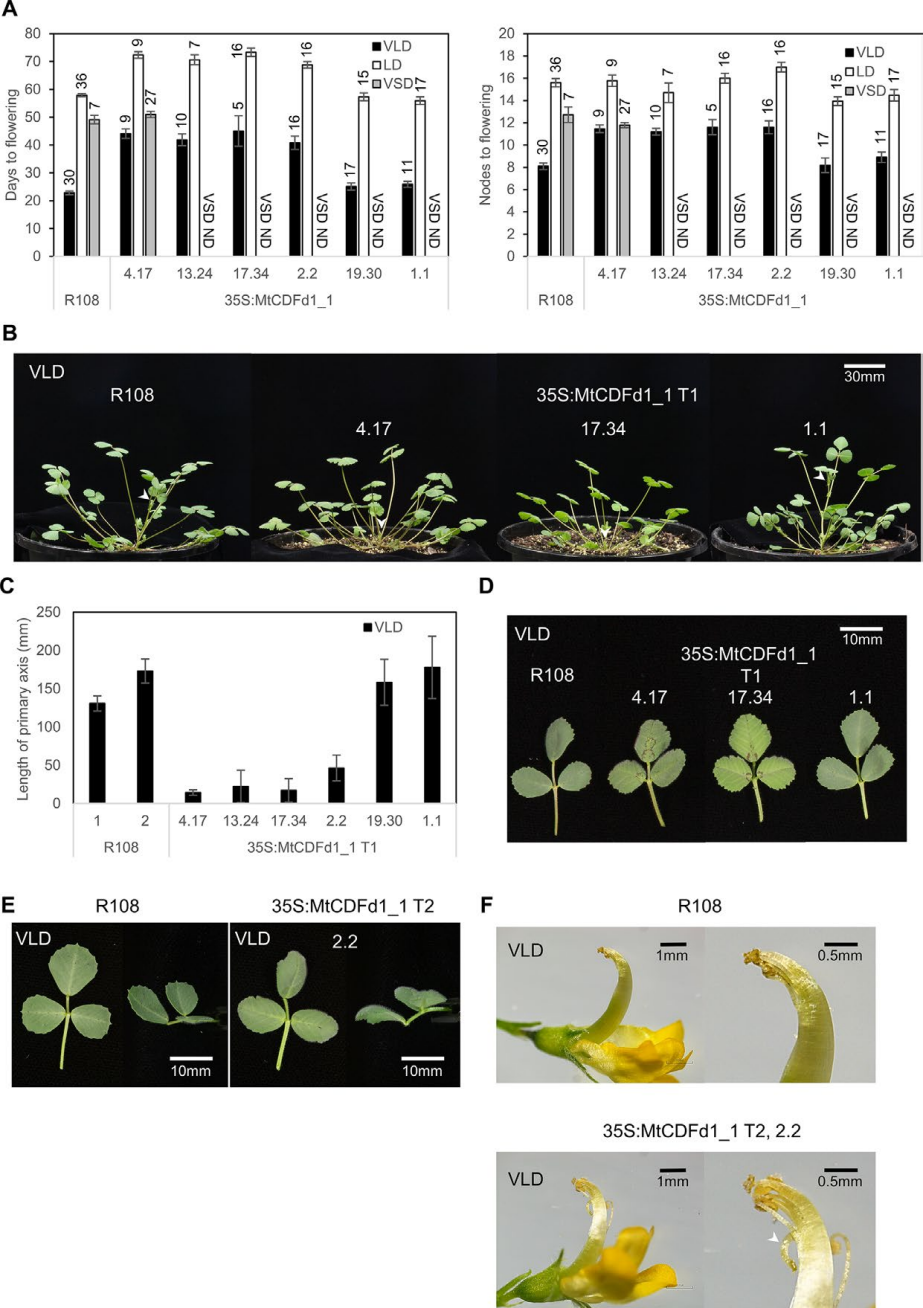
Overexpression of *MtCDFd1_1* in *Medicago* results in late flowering and reduced primary axis elongation. **(A)** Flowering time of six independent *35S:MtCDFd1_1 Medicago* R108 transgenic lines and R108 wild type in different conditions [vernalized long day (VLD), long day (LD), and vernalized short day (VSD)]. Either T_1_ or T_2_ generation plants were scored; data from different generations were not combined. Sample sizes are indicated above each bar. Flowering time was presented as the mean number of days, or the number of nodes on the primary axis when the first floral bud was observed ( ± t.SE 0.05) for each of the six independent transgenic lines and R108 control. ND meant that flowering time was not done under VSD. **(B)** Photographs of T_1_*35S:MtCDFd1_1* plants on day 29 under VLD. White arrows indicate the tip of the primary axis. **(C)** Mean length of the primary axis of the six independent T_1_ generation lines (41–50 days old) in VLD. The average primary axis length of each line was presented as ± t.SE (0.05), *n* = 5–10. The control line, R108-1, was planted and measured at the same time as lines 4.17, 13.24, 17.34, 19.30, and 1.1, while R108-2 was planted alongside line 2.2. **(D)** Photographs of 63-day-old fully expanded trifoliate leaves from different T_1_ plants and R108 in VLD. Trifoliate leaves photographed, from the top and from the side **(E)**, and flower **(F)** comparisons between R108 and *35S:MtCDFd1_1* line 2.2. Photographs were taken when VLD R108 and *35S:MtCDFd1_1* plants were 71 and 86 days old, respectively. The white arrow indicates the abnormal curled-down pistil in the *35S:MtCDFd1_1* line compared to wild-type plants.

We overexpressed *MtCDFd1_1* in *Medicago* to assay the effect this would have on flowering time. After co-cultivation of *Medicago* wild-type R108 leaf disks with *Agrobacterium* carrying the *35S:MtCDFd1_1* transgene, we selected six independent T_0_ transformants. T_1_ or T_2_ progeny was scored for flowering time in two photoperiodic conditions, with and without prior vernalization (VLD, LD, and VSD; **Figure 3A**).

As expected, VLD most strongly accelerated the flowering of R108 wild-type plants, out of the three conditions tested (**Figure 3A**). In contrast, most of the transgenic lines (four of six lines: 4.17, 13.24, 17.34, and 2.2) showed delayed flowering in VLD, in both days and nodes at flowering (**Figure 3A**). In LD, the same four lines showed later flowering than R108 in days to flowering. However, only line 2.2 flowered marginally later in nodes, indicating overall a much weaker flowering time phenotype in LD.

**Figure 4:**
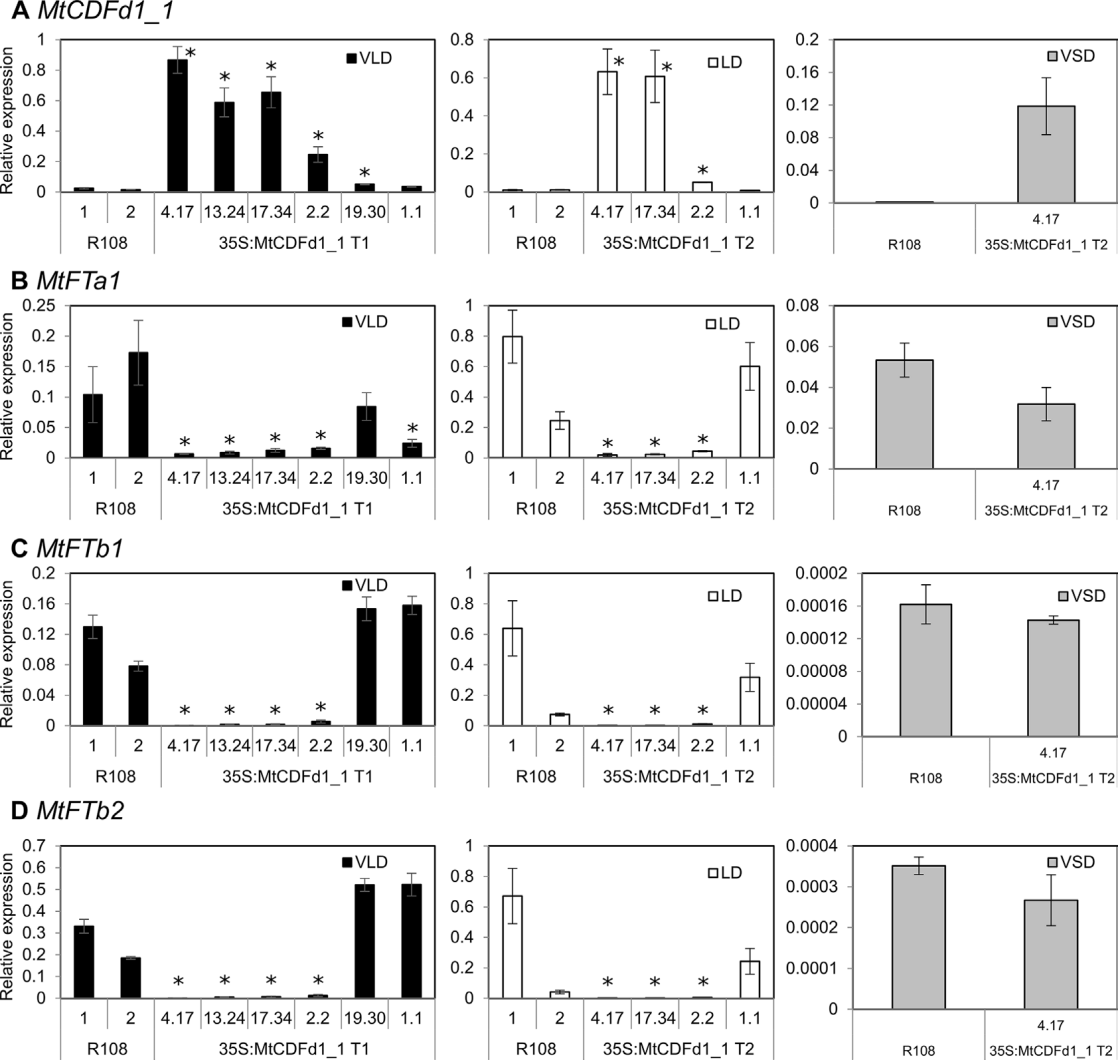
*MtCDFd1_1* overexpression in *Medicago* reduces *MtFTa1*, *MtFTb1*, and *MtFTb2* transcript levels. **(A**–**D)** Expression of *MtCDFd1_1*, *MtFTa1*, *MtFTb1*, and *MtFTb2* in the *35S:MtCDFd1_1 Medicago* R108 transgenic lines in vernalized long day (VLD), long day (LD), and vernalized short day (VSD). Data were derived from fully expanded trifoliate leaves harvested on days 14 and 15 (VLD), day 46 (LD), and day 43 (VSD) at ZT4. Gene expression levels are means ± SE of three biological replicates, normalized to *PP2A*. Data were presented relative to the highest value of a gene across the three growth conditions. In VLD, R108-1 was grown at the same time as lines 4.17, 13.24, 17.34, 19.30, and 1.1, while R108-2 was grown with line 2.2. In LD, R108-1 was grown at the same time as lines 17.34 and 1.1, while R108-2 with lines 4.17 and 2.2. All plants grown in VLD were T_1_ generation, while T_2_ populations were grown in LD and VSD. Asterisks indicate transgenic lines with significantly different expression from R108 [multiple pairwise comparisons adjusted for false discovery rate (FDR); α = 0.05].

Line 4.17 was then chosen as the representative transgenic line to test in VSD conditions. It had previously shown no phenotypic differences in VLD conditions from three other independent transgenic lines (13.24, 17.34, and 2.2) that also strongly overexpressed *MtCDFd1_1* (**Figure 4A**). Line 4.17 flowering time was not statistically significantly different to R108 in VSD, indicating that *35S:MtCDFd1_1* did not confer late flowering relative to wild type in VSD conditions in this line. Additionally, we observed that line 4.17 flowered at a similar time in VSD and VLD. In summary, while *35S:MtCDFd1_1* caused late flowering in VLD compared to wild type, it had no significant effect in VSD in line 4.17, resulting in day-neutral flowering. Thus, flowering time analysis in VSD was not pursued further.

Wild-type R108 plants grown in VLD conditions also typically show elongation of the primary shoot axis at the time of flowering. Therefore, as might be expected from their late-flowering phenotype, the four late-flowering transgenic lines had a shorter primary axis in VLD compared to R108. This was observed at the flowering of R108 and the *35S:MtCDFd1_1* plants (**Figures 3B, C**).

In addition, the leaves of later-flowering transgenic plants were sometimes paler in color than R108 and the transgenic plants that did not flower late (**Figure 3D**). In the later stage of plant growth, they had trifoliate leaves that curved down (epinastic) while R108 leaves curved upwards (**Figure 3E**). Some late-flowering transgenic plants displayed sterility. This was likely because the top of the pistil was curled down, causing the stigma to be away from anthers, leading to failure in pollination (**Figure 3F**).

In summary, four of the six independent lines carrying the *35S:MtCDFd1_1* transgene showed delayed flowering and changes to architecture including shorter primary stems, leaf curling, and infertility in VLD conditions.

### *MtCDFd1_1* Overexpression Is Negatively Correlated With Transcript Levels of *MtFT-Like* Genes but Not *MtCOL* Genes

To investigate the basis of the late-flowering phenotypes observed in the *35S:MtCDFd1_1* transgenic lines (**Figure 3A**), we analyzed gene expression by qRT-PCR (**Figure 4**). The genes assayed were *MtCDFd1_1* and the three LD-induced *MtFT* genes, which are expressed at higher levels in VLD than in VSD: *MtFTa1*, *MtFTb1*, and *MtFTb2* (Laurie et al., 2011). *MtFTa1* has been shown to accelerate flowering when overexpressed in *Medicago*, while loss-of-function mutants show late flowering compared to wild type, particularly in VLD conditions (Laurie et al., 2011; Jaudal et al., 2019).

In VLD, *35S:MtCDFd1_1* transcript levels in the four late-flowering lines (4.17, 13.24, 17.34, and 2.2) were significantly higher compared to those in R108 controls (**Figure 4A**). However, *MtCDFd1_1* expression in the fifth line was only very weakly elevated, while the sixth line, 1.1, was not significantly different from R108. These latter two lines, 19.30 and 1.1, also flowered at a similar time to R108 (**Figure 3A**).

The increased expression of *MtCDFd1_1* in VLD observed in the four transgenic lines 4.17, 13.24, 17.34, and 2.2 (**Figure 4A**) correlated with significantly lower abundance of *MtFTa1*, *MtFTb1*, and *MtFTb2* transcripts (**Figures 4B–D**) and late flowering (**Figure 3A**) in those lines compared to wild-type R108 plants.

**Figure 5:**
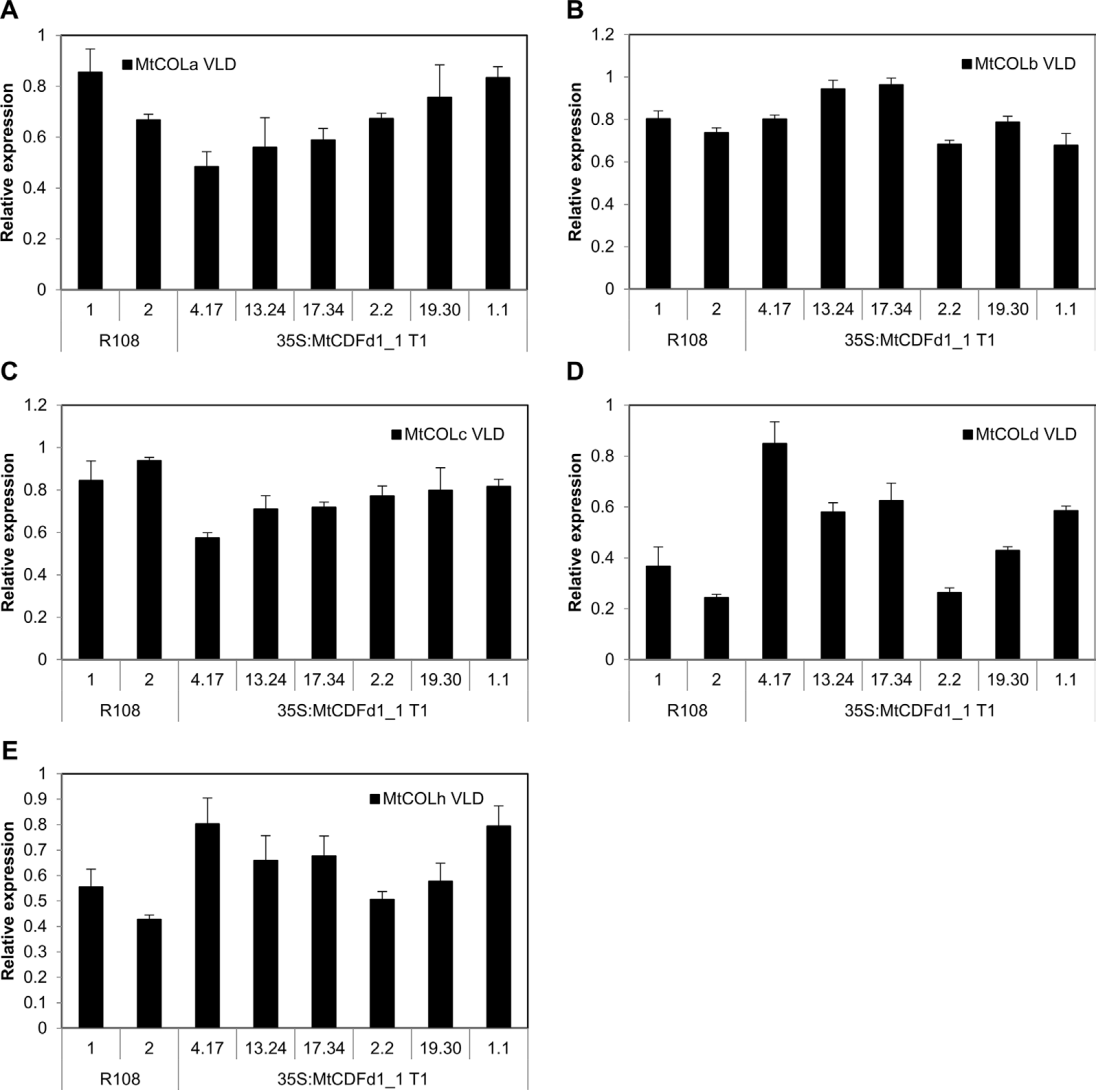
Overexpression of *MtCDFd1_1* in *Medicago* does not reduce *COL* gene expression. **(A**–**E)** Relative gene expression of five Medicago *COL* genes in vernalized long day (VLD) in *35S:MtCDFd1_1* lines. Data were derived from fully expanded trifoliate leaves harvested from T_1_ plants on days 14 and 15 at ZT4. Gene expression levels were means of three biological replicates ± SE, normalized to *PP2A*. Data were presented relative to the sample with the highest expression. R108-1 was grown at the same time as lines 4.17, 13.24, 17.34, 19.30, and 1.1, while R108-2 was grown with line 2.2.

In contrast, qRT-PCR analysis of five *MtCOL* genes (*MtCOLa–MtCOLd* and *MtCOLh*; **Figure 5**) indicates that there is no consistent change to the expression of these genes in the four *Mt MtCDFd1_1* overexpression lines (4.17, 13.24, 17.34, and 2.2) compared to R108 and the two remaining transgenic lines that do not overexpress *MtCDFd1_1* (19.30 and 1.1).

In LD, a subset of lines was tested for gene expression. Like in VLD, overexpression of *MtCDFd1_1* correlated with significantly reduced expression of *MtFTa1*, *MtFTb1*, and *MtFTb2* (**Figure 4**).

In VSD, no significant difference could be seen in the expression of *MtFTa1* in representative *MtCDFd1_1* overexpressing line 4.17 relative to R108 (**Figure 4B**). This is consistent with the absence of a flowering time phenotype in this transgenic line relative to R108 in VSD. *MtFTb1* and *MtFTb2* transcript levels were barely detectable in VSD in the transgenic line or R108 (**Figures 4C, D**) as expected (Laurie et al., 2011). Thus, gene expression analysis in VSD was not pursued further.

### Flowering Time and Gene Expression in *35S:MtCDFd1_1/Mtfta1* Homozygous Lines

**Figure 6:**
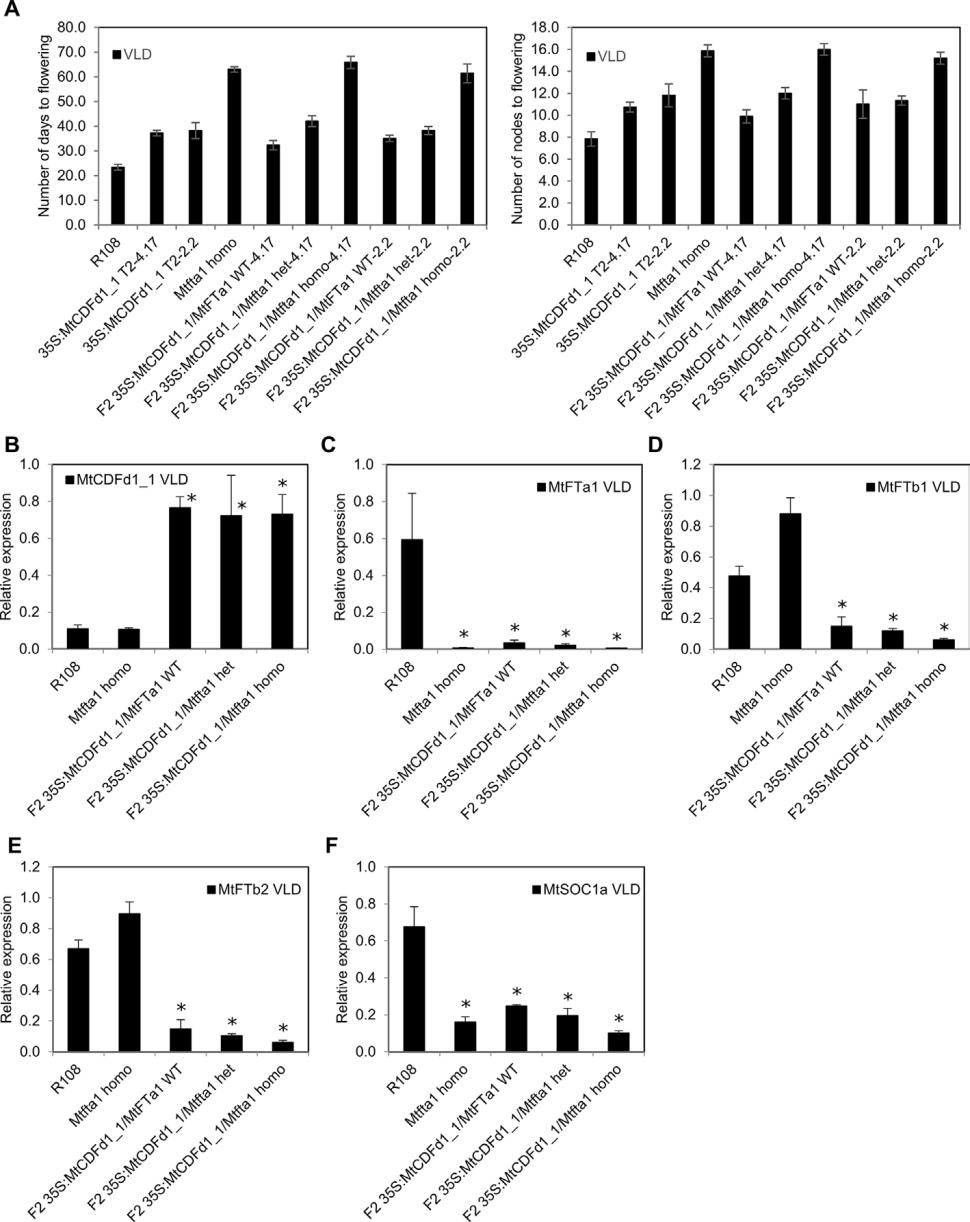
Analysis of an F_2_ population from a cross between late-flowering *Medicago* plants overexpressing *MtCDFd1_1* and the late-flowering *Mtfta1* R108 mutant. **(A)** Flowering time for each genotype in a segregating F_2_ population (*n* = 72) derived from a cross between the *Mtfta1* mutant and transgenic plants overexpressing *MtCDFd1_1* is presented as either the mean number of days or the number of nodes on the primary axis when the first floral bud was observed ± t.SE (0.05). Homo is homozygous for the *Mtfta1* mutation, and het is heterozygous for the *Mtfta1* mutation. One wild-type F_2_ segregant plant (without the *35S:MtCDFd1_1* transgene and wild type for *MtFTa1*) was obtained. It flowered at 26 days and eight nodes, similar to wild-type R108. **(B**–**F)** Relative gene expression in 35S:MtCDFd1_1 F2 plants, with or without the Mtfta1 mutation of MtCDFd1_1, MtFTa1, MtFTb1, MtFTb2 and MtSOC1a in vernalized long day (VLD). Data were derived from fully expanded trifoliate leaves harvested on day 23 at ZT4. Three biological samples each consisting of leaves from three plants were harvested per genotype. Gene expression levels were means of the three biological replicates ± SE, normalized to *PP2A*. Data were presented relative to the highest value of that specific gene. Asterisks indicate genotypes with significantly different expression from R108 [multiple pairwise comparisons adjusted for false discovery rate (FDR); α = 0.05].

*MtFTa1* is a strong promoter of *Medicago* flowering, particularly in VLD conditions (Laurie et al., 2011). This suggests that the delayed flowering in the *35S:MtCDFd1_1* plants in VLD might be due to the reduced average *MtFTa1* expression we observed. Therefore, to analyze the interaction between *35S:MtCDFd1_1* and *MtFTa1*, two late-flowering *35S:MtCDFd1_1* lines, 4.17 and 2.2, were crossed with the late-flowering *Mtfta1* mutant and the resulting F_2_ populations scored in VLD (**Figure 6A**).

*35S:MtCDFd1_1/Mtfta1* homozygous F_2_ plants flowered ∼1 month later than *35S:MtCDFd1_1* lines homozygous for wild-type *MtFTa1*, but at a similar time to *Mtfta1* homozygous mutant plants. Thus, no additive effect was observed in *35S:MtCDFd1_1* on the late flowering already conferred by the *Mtfta1* homozygous mutation in VLD.

As previously observed in the four late-flowering *35S:MtCDFd1_1* transgenic plants (lines 4.17, 13.24, 17.34, and 2.2, **Figure 4**), the presence of the *35S:MtCDFd1_1* transgene correlated with significantly lower transcript levels of *MtFTa1*, *MtFTb1*, and *MtFTb2* compared to R108 (**Figures 6B–E**).

We also analyzed the expression of *MtSOC1a* (**Figure 6F**), a *SOC1-like* gene which promotes flowering and primary stem growth and whose expression is partly dependent on *MtFTa1* (Fudge et al., 2018; Jaudal et al., 2018). Plants with the *35S:MtCDFd1_1* transgene and wild type for *MtFTa1* showed a statistically significant, moderate decrease (∼2.7-fold) in average *MtSOC1a* transcript levels compared to wild-type R108 plants.

## Discussion

While the photoperiodic pathways in *Medicago* and pea promote flowering through LD-induced *FT* genes such as *FTa1*, in contrast to *Arabidopsis*, they appear to act in a *CO*-independent manner. To test whether *MtCDF* genes regulate *Medicago* photoperiodic flowering time, we analyzed the expression and function of members of the *MtCDF* clade with a focus on *MtCDFd1_1*. Our work on the *MtCDF*s has revealed similarities and differences between *Medicago* and the well-characterized *Arabidopsis* system and indicates how *MtCDF*s may contribute to *Medicago* flowering time control.

*MtCDF* genes, *MtCDFd1_1* (here) and *MtCDFc2-1* and *MtCDFb2* (Thomson et al., 2019), showed a diurnal cycle of expression, with peak transcript levels at or near dawn, which was similar to the best characterized *AtCDF*s that regulate flowering time (*AtCDF1-3,5*). We also observed that overexpression of *MtCDFd1_1* in *Medicago* caused VLD plants to flower late, as if they had been grown in VSD, rendering the transgenic plants day neutral. These results are similar to those reported for the dominant pea mutation *late2/Pscdfc1* (Ridge et al., 2016) and for overexpression of *AtCDFs* in *Arabidopsis*. Thus, *MtCDF*s may normally function in wild-type plants predominantly to delay flowering in VSD.

*35S:MtCDFd1_1* appears to regulate flowering in *Medicago via* repressing *MtFTa1*, a known strong promoter of flowering in VLD (Laurie et al., 2011), but not *via MtCOL* genes. The transcript levels of the LD-induced genes *MtFTa1*, *MtFTb1*, *MtFTb2*, and *MtSOC1a* were significantly reduced in the *35S:MtCDFd1_1* transgenic plants, while five *MtCOL* genes were unaffected. Genetic analysis showed that *35S:MtCDFd1_1/Mtfta1* plants flowered no later than the later-flowering parent, *Mtfta1*. Thus, in VLD, *35S:MtCDFd1_1* influenced flowering in the same pathway as *MtFTa1*, and the late flowering of *35S:MtCDFd1_1* plants in VLD likely results from reduced *MtFTa1* gene expression. The short primary stem phenotype observed is also consistent with the repressive effect of *35S:MtCDFd1_1* on expression of *MtFTa1* and *MtSOC1a*, previously indicated to be important for stem elongation in VLD and LD conditions (Laurie et al., 2011; Jaudal et al., 2018).

What might be the role of the other two *MtFT-like* genes, *MtFTb1* and *MtFTb2*, whose expression is also strongly reduced by *35S:MtCDFd1_1*? The *35S:MtCDFd1_1/Mtfta1* plants show no additional delay to flowering time, beyond that conferred by the *Mtfta1* mutation in VLD conditions, and as previously reported (Laurie et al., 2011), *MtFTb1* and *MtFTb2* expression is not affected by the single *Mtfta1* mutation. Overall, these results indicate that neither *MtFTb1* nor *MtFTb2* has non-redundant roles in *Medicago* flowering time in VLD. It is possible they may affect flowering *via* regulating *MtFTa1*, but testing this awaits the identification of single and double *MtFTb1/2* mutant plants.

While no *MtCDF Tnt1* insertion mutant plants had a flowering time phenotype, this is overall consistent with *Arabidopsis CDF* single mutants and is likely due to redundancy in function between the genes (Fornara et al., 2009). On the other hand, five genes (*MtCDFd1_1*, *MtCDFa2*, *MtCDFc1*, *MtCDFd1_3*, and *MtCDFe*), out of the 11 tested, stood out for their ability to cause late flowering when overexpressed in *Arabidopsis*. It is possible that sequence variation within key MtCDF functional domains, or their absence, could affect the other MtCDFs’ ability to interact with potential binding partners or target genes and regulate flowering time. For example, differential susceptibility to the *Arabidopsis* FKF1/GI protein degradation system may affect MtCDFs’ ability to repress flowering and could help explain some of the variation in flowering times observed between the different genes (Kloosterman et al., 2013; Ridge et al., 2016). On the other hand, it is possible that the inability of the other *MtCDF* genes tested to affect *Arabidopsis* flowering time was due to the differences in transgene expression levels.

In our case, *35S:MtCDFd1_1* strongly represses flowering, and its predicted protein lacks the predicted GI- and FKF1-binding domains. This provides some indication that MtCDFd1_1 protein may not be targeted for degradation by the endogenous FKF1/GI system in *Arabidopsis* or *Medicago*, suggesting an alternative method of regulation of its activity in LD from the AtCDF system. On the other hand, MtCDFc1 and its predicted pea ortholog PsCDFc1 (Ridge et al., 2016) do interact with AtFKF1 in yeast two-hybrid assays but have different effects on flowering in *Arabidopsis*. In our experiments, *35S:MtCDFc1* strongly delayed *Arabidopsis* flowering, while *35S:PsCDFc1* was reported not to (Ridge et al., 2016). This indicates that other differences in sequence may be important, or perhaps differences in cultivation or levels of expression in the transgenic plants may be responsible.

Apart from a delayed transition to flowering, other phenotypes were seen in multiple *35S:MtCDF* transgenic *Arabidopsis* plants implicating *MtCDF*s in a variety of plant processes that extend beyond involvement in photoperiodic regulation (Corrales et al., 2014, Corrales et al., 2017). In plants such as *Arabidopsis* and tomato, *CDF* genes also modulate other processes such as abiotic stress tolerance (Corrales et al., 2014, Corrales et al., 2017). In addition, a different photoperiodic process, namely, SD-induced tuber development, is regulated by *StCDF1* in *Solanum tuberosum* L. (potato; Kloosterman et al., 2013). The abnormal phenotypes we observed included an upright rosette leaf stature with rigid long-handle spoon-shaped curved leaves, which may indicate effects on hormone homeostasis (e.g. Sun et al., 2010). Interestingly, in addition to late flowering in some independent *35S:MtCDFd1_3* lines, the older leaves of some of the plants developed spotty lesions, perhaps indicative of effects on senescence or cell death and/or disease resistance processes (Lorrain et al., 2003).

Overall, our results expand the understanding of the features and functions of members of the MtCDF clade. *MtCDF* genes are implicated as regulators of the *Medicago* photoperiodic pathway, where they are likely to have overlapping functions in wild-type plants probably by repressing flowering in VSD conditions. In terms of mechanism, the absence of an effect of overexpression of *MtCDFd1_1* in transgenic lines (4.17, 13.24, 17.34, and 2.2) on the expression of five *MtCOL* genes (**Figure 5**), but strong repression of LD-induced *MtFT* genes compared to R108 (**Figure 4**), adds further support to the idea that *MtCDF*s may function in a photoperiod pathway that is independent of *CO*. This is consistent with work in pea (Ridge et al., 2016). Future work to determine the function of *MtCDF*s and to overcome the challenges of functional redundancy will focus on generating plants carrying mutations in multiple *MtCDF* genes using the CRISPR–Cas9 system in *Medicago* (Meng et al., 2017; Curtin et al., 2018). In addition, since there is direct regulation of *FT* by AtCDF1 (Song et al., 2012), direct interactions of MtCDFs with the LD-induced *MtFT-like* genes could be tested to examine if this is conserved in legumes.

## Data Availability

All datasets generated for this study are included in the manuscript and the supplementary files.

## Author Contributions

LZ, AJ, MK-P, GT, TK, CP, and MJ performed the experiments, LZ, AJ, GT, and JP prepared the figures. JW and KM provided the *Medicago Tnt1* insertion lines. JP conceived the project and wrote the article with contributions from AJ, LZ, MJ, and GT.

## Funding

The research was funded by the New Zealand Foundation for Research Science and Technology (www.msi.govt.nz/) contract number C10X0816 MeriNET and the New Zealand Marsden Fund (www.royalsociety.org.nz/programmes/funds/marsden/) contract 14-UOA-125. The development of the *Medicago Tnt1* insertion lines and reverse genetics screenings were supported by the National Science Foundation, USA (DBI 0703285 and IOS-1127155), and Noble Research Institute, LLC.

## Conflict of Interest Statement

The authors declare that the research was conducted in the absence of any commercial or financial relationships that could be construed as a potential conflict of interest.
