# Supplementary material for: Overexpression of Medicago MtCDFd1_1 Causes Delayed Flowering in Medicago via Repression of MtFTa1 but Not MtCO-Like Genes
Source: Front Plant Sci. 2019 Sep 19;10:1148. doi: 10.3389/fpls.2019.01148 (PMC6761483; doi:10.3389/fpls.2019.01148)
Supplement: Supplementary file 1 [file DataSheet_1.pdf]

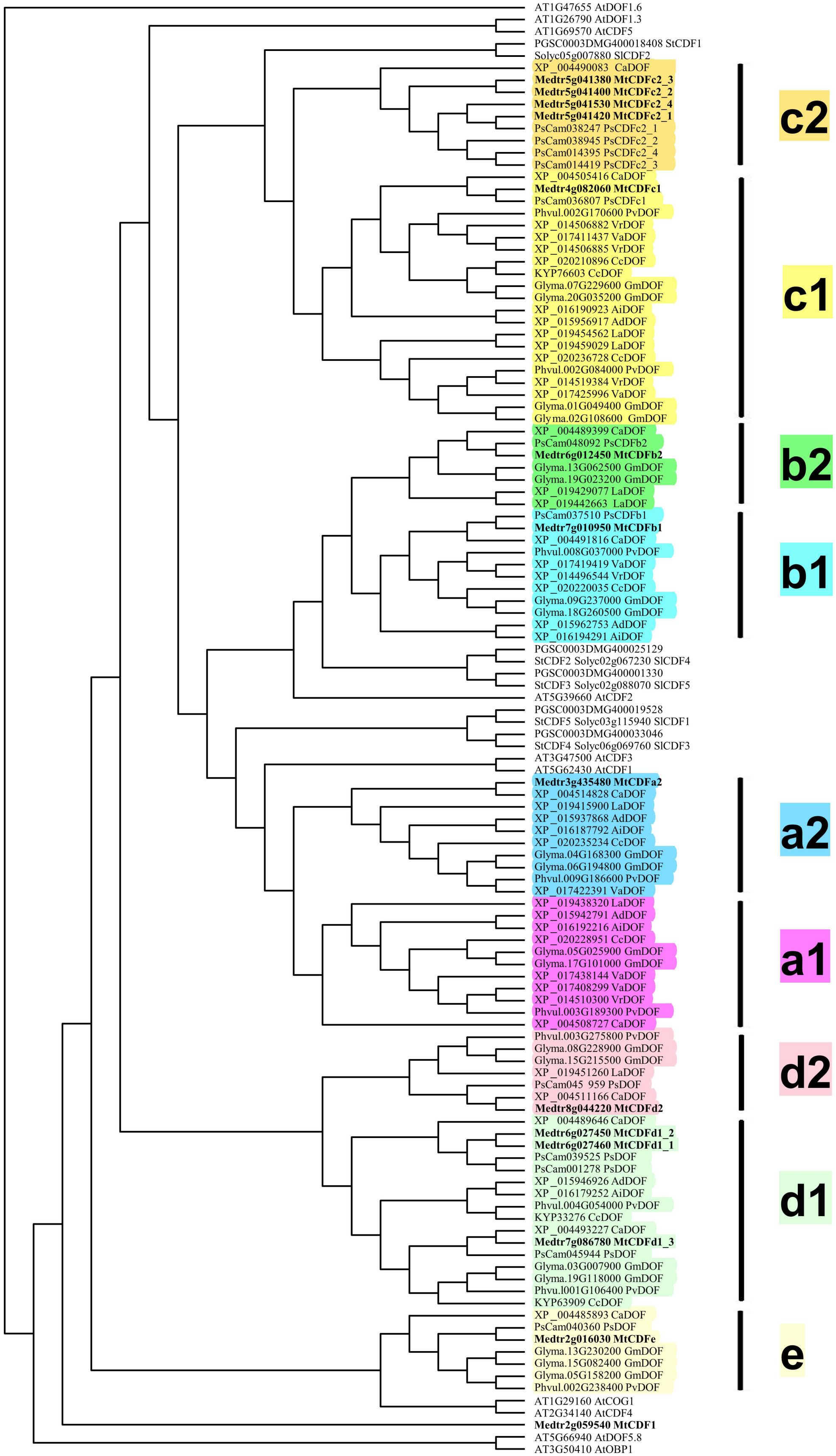
AT1G47655 AtDOF1.6
AT1G26790 AtDOF1.3
AT1G69570 AtCDF5
PGSC0003DMG400018408 StCDF1
Solyc05g007880 SlCDF2
XP _004490083 CaDOF
Medtr5g041380 MtCDFc2_3
Medtr5g041400 MtCDFc2_2
Medtr5g041530 MtCDFc2_4
Medtr5g041420 MtCDFc2_1
PsCam038247 PsCDFc2_1
PsCam038945 PsCDFc2_2
PsCam014395 PsCDFc2_4
PsCam014419 PsCDFc2_3
XP _004505416 CaDOF
Medtr4g082060 MtCDFc1
PsCam036807 PsCDFc1
Phvul.002G170600 PvDOF
XP _014506882 VrDOF
XP _017411437 VaDOF
XP _014506885 VrDOF
XP _020210896 CcDOF
KYP76603 CcDOF
Glyma.07G229600 GmDOF
Glyma.20G035200 GmDOF
XP _016190923 AiDOF
XP _015956917 AdDOF
XP _019454562 LaDOF
XP _019459029 LaDOF
XP _020236728 CcDOF
Phvul.002G084000 PvDOF
XP _014519384 VrDOF
XP _017425996 VaDOF
Glyma.01G049400 GmDOF
Glyma.02G108600 GmDOF
XP _004489399 CaDOF
PsCam048092 PsCDFb2
Medtr6g012450 MtCDFb2
Glyma.13G062500 GmDOF
Glyma.19G023200 GmDOF
XP _019429077 LaDOF
XP _019442663 LaDOF
PsCam037510 PsCDFb1
Medtr7g010950 MtCDFb1
XP _004491816 CaDOF
Phvul.008G037000 PvDOF
XP _017419419 VaDOF
XP _014496544 VrDOF
XP _020220035 CcDOF
Glyma.09G237000 GmDOF
Glyma.18G260500 GmDOF
XP _015962753 AdDOF
XP _016194291 AiDOF
PGSC0003DMG400025129
StCDF2 Solyc02g067230 SlCDF4
PGSC0003DMG400001330
StCDF3 Solyc02g088070 SlCDF5
AT5G39660 AtCDF2
PGSC0003DMG400019528
StCDF5 Solyc03g115940 SlCDF1
PGSC0003DMG400033046
StCDF4 Solyc06g069760 SlCDF3
AT3G47500 AtCDF3
AT5G62430 AtCDF1
Medtr3g435480 MtCDFa2
XP _004514828 CaDOF
XP _019415900 LaDOF
XP _015937868 AdDOF
XP _016187792 AiDOF
XP _020235234 CcDOF
Glyma.04G168300 GmDOF
Glyma.06G194800 GmDOF
Phvul.009G186600 PvDOF
XP _017422391 VaDOF
XP _019438320 LaDOF
XP _015942791 AdDOF
XP _016192216 AiDOF
XP _020228951 CcDOF
Glyma.05G025900 GmDOF
Glyma.17G101000 GmDOF
XP _017438144 VaDOF
XP _017408299 VaDOF
XP _014510300 VrDOF
Phvul.003G189300 PvDOF
XP _004508727 CaDOF
Phvul.003G275800 PvDOF
Glyma.08G228900 GmDOF
Glyma.15G215500 GmDOF
XP _019451260 LaDOF
PsCam045 959 PsDOF
XP _004511166 CaDOF
Medtr8g044220 MtCDFd2
XP 004489646 CaDOF
Medtr6g027450 MtCDFd1_2
Medtr6g027460 MtCDFd1_1
PsCam039525 PsDOF
PsCam001278 PsDOF
XP _015946926 AdDOF
XP _016179252 AiDOF
Phvul.004G054000 PvDOF
KYP33276 CcDOF
XP _004493227 CaDOF
Medtr7g086780 MtCDFd1_3
PsCam045944 PsDOF
Glyma.03G007900 GmDOF
Glyma.19G118000 GmDOF
Phvu.l001G106400 PvDOF
KYP63909 CcDOF
XP _004485893 CaDOF
PsCam040360 PsDOF
Medtr2g016030 MtCDFe
Glyma.13G230200 GmDOF
Glyma.15G082400 GmDOF
Glyma.05G158200 GmDOF
Phvul.002G238400 PvDOF
AT1G29160 AtCOG1
AT2G34140 AtCDF4
Medtr2g059540 MtCDF1
AT5G66940 AtDOF5.8
AT3G50410 AtOBP1
c2
c1
b2
b1
a2
a1
d2
d1
e

Supplementary Figure S1: Phylogenetic tree of CDF-like proteins from *Medicago,* other legumes, tomato, potato and Arabidopsis. The tree was constructed with full length amino acid sequences using the neighbour-joining algorithm implemented in PAUP* (version 4.0). Clades (a-c) of legume proteins have been named as described in Ridge et al. (2016). Additional nomenclature extended to clades d & e. Species included are; Ad - *Arachis duranensis,* Ai - *Arachis ipaensis,* At - *Arabidopsis thaliana, Ca - Cicer arietinum,* Cc - *Cajanus cajan,* Gm - *Glycine max,* La - *Lupinus augustifolius,* Mt - *Medicago truncatula,* Ps - *Pisum sativa.* Pv - *Phaseolus vulgaris,* Sl - *Solanum lycopersicum,* St - *Solanum tuberosum,* Va - *Vigna angularis* and Vr - *Vigna radiata.*

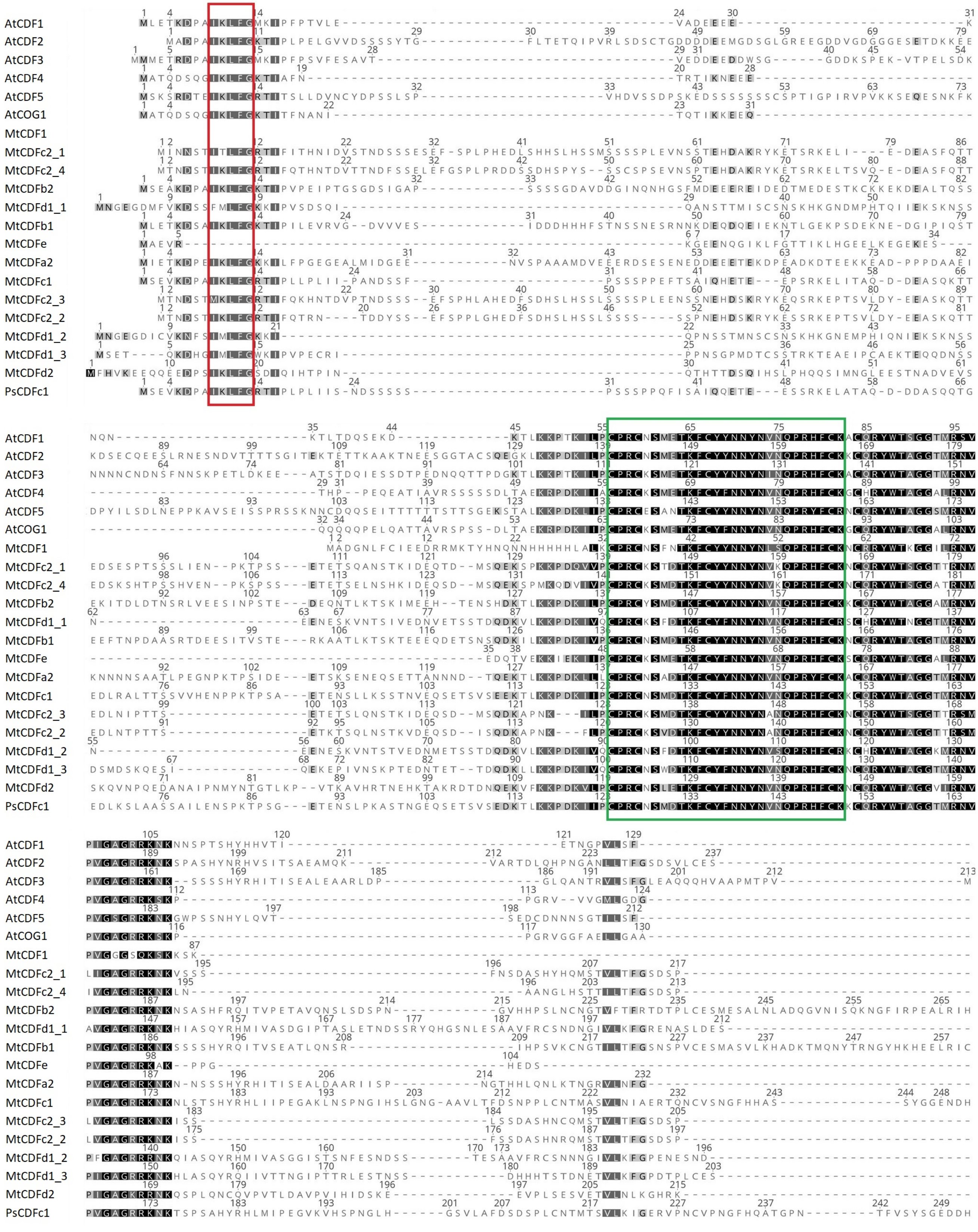

AtCDF1
AtCDF2
AtCDF3
AtCDF4
AtCDF5
AtCOG1
MtCDF1
MtCDFc2_1
MtCDFc2_4
MtCDFb2
MtCDFd1_1
MtCDFb1
MtCDFe
MtCDFa2
MtCDFc1
MtCDFc2_3
MtCDFc2_2
MtCDFd1_2
MtCDFd1_3
MtCDFd2
PsCDFc1

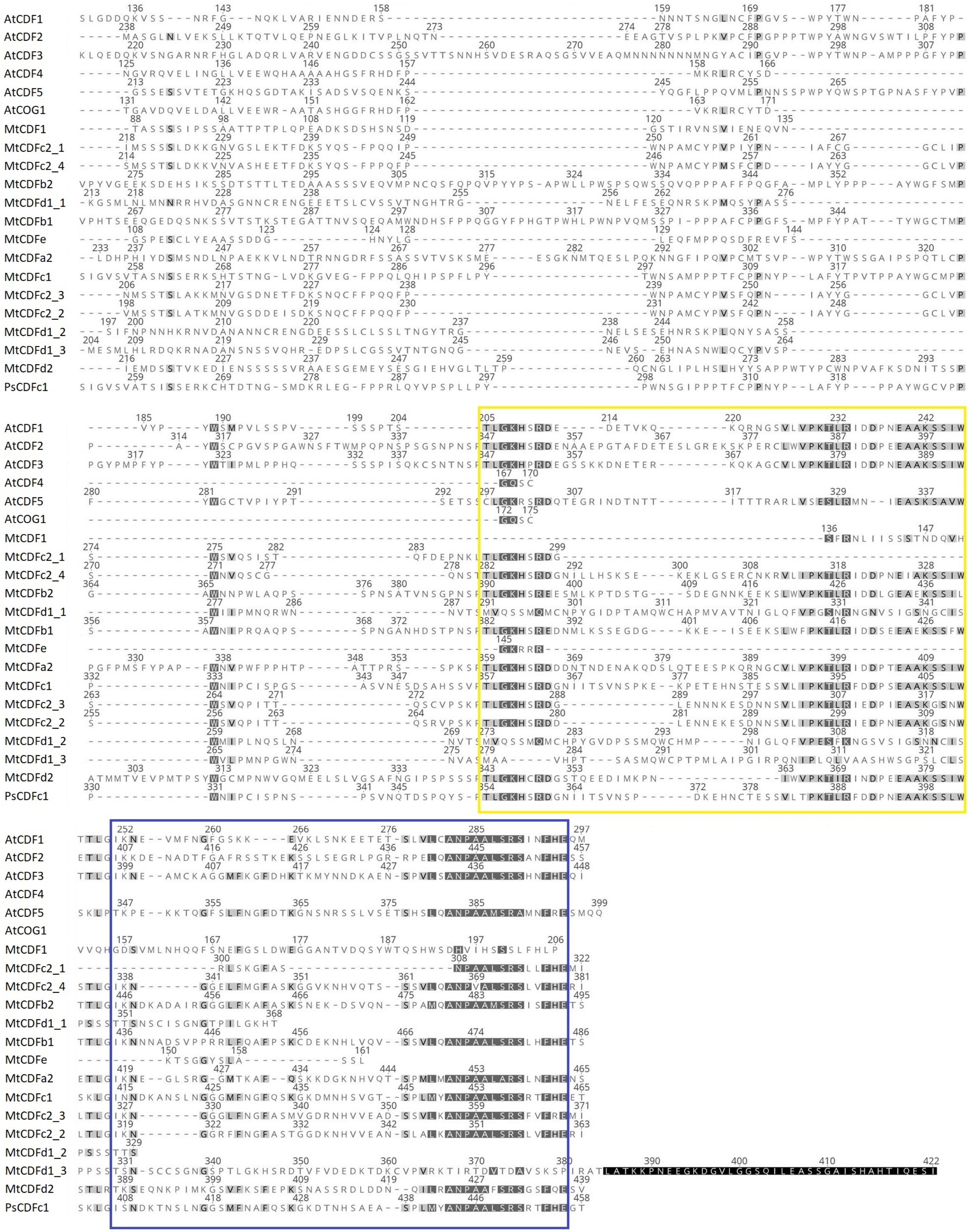
AtCDF1
AtCDF2
AtCDF3
AtCDF4
AtCDF5
AtCOG1
MtCDF1
MtCDFc2_1
MtCDFc2_4
MtCDFb2
MtCDFd1_1
MtCDFb1
MtCDFe
MtCDFa2
MtCDFc1
MtCDFc2_3
MtCDFc2_2
MtCDFd1_2
MtCDFd1_3
MtCDFd2
PsCDFc1

Supplementary Figure S2: Alignment of Medicago, Arabidopsis and pea CDF protein sequences showing the highly conserved DOF domain (green box) present in all the proteins and three other domains present in most of the CDF proteins, the N- terminal TOPLESS (TPL) interacting domain (red box) and two C-terminal domains, a putative GI-interacting domain (yellow box) and a FKF1-interacting region (blue).

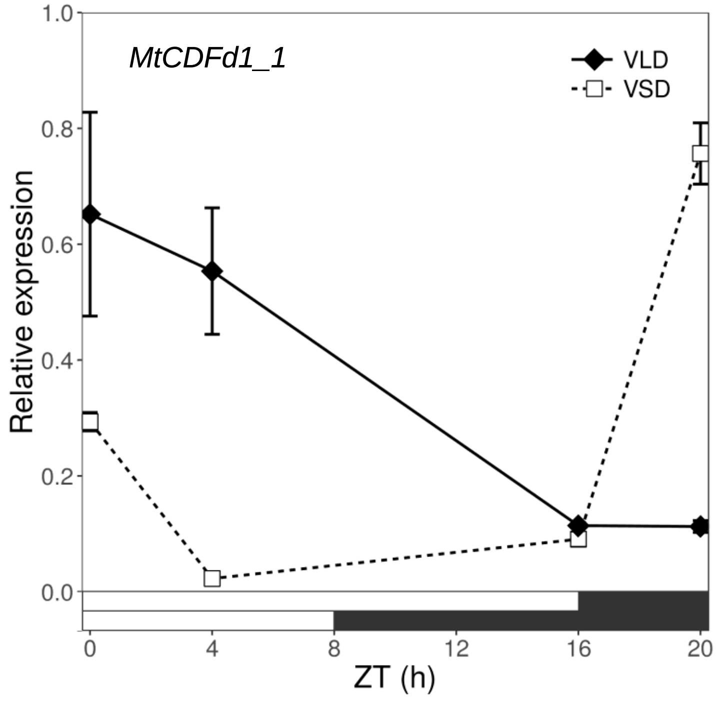

Supplementary Figure S3. *MtCDFd1_1* gene transcript levels have a diurnal cycle. Gene expression of *MtCDFd1_1* in a Jester diurnal time course in VSD and VLD. Results are presented as averages ± SE of three biological replicates at each time point and normalised to Medicago *PROTEIN PHOSPHATASE 2A* (*PP2A)*. Data are presented relative to the highest value observed overall. Bars underneath each graph show the length of light and dark conditions for LD (16 h light/8 h dark; upper) and SD (8 h light/16 dark; lower). ZT (zeitgeber time) indicates hours after dawn.

Each replicate consists of two fully-expanded trifoliate leaves from vernalised (V) wild type Jester plants grown in SD or LD.

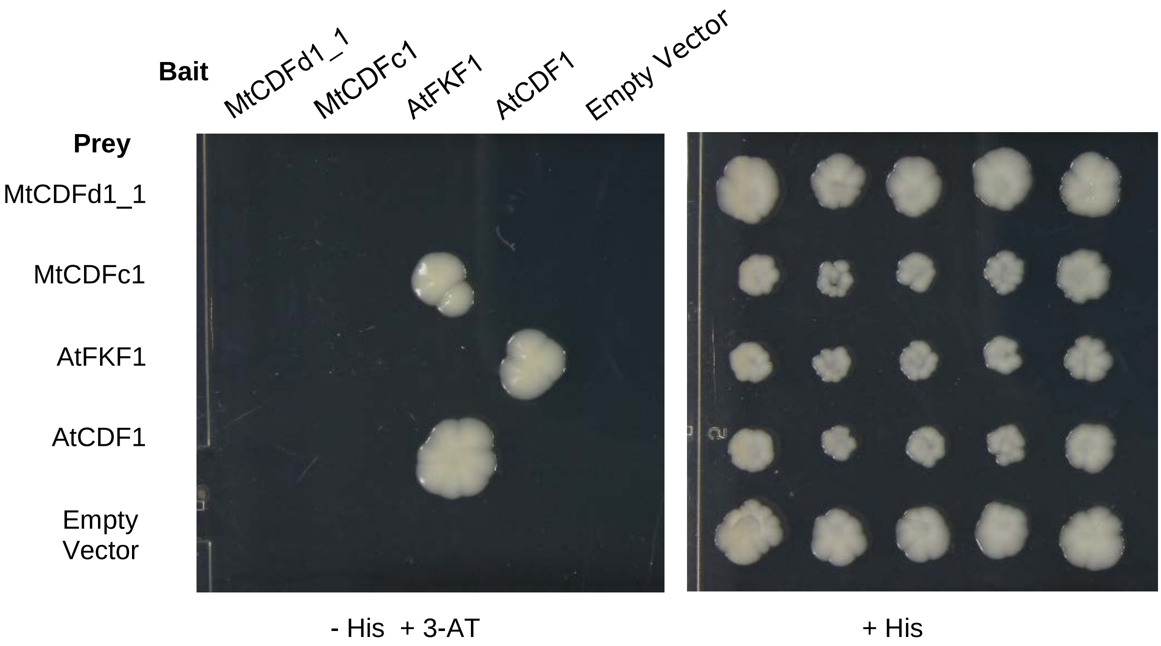

Supplementary Figure S4. *MtCDFd1_1* does not interact with AtFKF1 in yeast two hybrid assays. (**B**) Diploid yeast colonies containing prey and bait constructs from *MtCDFd1_1*, *MtCDFc1*, AtCDF1 and FKF1 or empty vector controls, were grown on medium selecting for protein-protein interactions, lacking His with 2mM 3-AT (left) or on medium with His (right). Colonies were allowed to develop over 11 days at 28°C. The vectors were pDEST22 (prey vector with the GAL4 activation domain) and pDEST32 (bait vector with GAL4 binding domain). Vectors containing inserts encoding full length CDF proteins were tested while the fragment encoding the KELCH-repeat region of AtFKF1 (amino acids 284 to 619) was used. The positive control interaction was between AtCDF1 and AtFKF1; AtCDF1, as either bait or prey, interacted with AtFKF1 (Imaizumi et al., 2005; Ridge et al., 2016). One of the Medicago CDFs, MtCDFc1 interacted with AtFKF1 only in one direction, when it was the prey. However, as predicted from the lack of conservation of the C-terminal domains with predicted GI and FKF1 binding functions, there was no evidence of interaction between MtCDFd1_1 and AtFKF1. Similar results were obtained with a second experiment using independent colonies.

Supplementary Table S1: Summary of the results of screening of *MtCDF Tnt1* insertion mutants.

Medicago gene ID[1]	Gene Names[2]	*Thomson et al.* Nomenclature[4]	*Tnt1* line	*Tnt1* position	Fl. time LD[+]		Fl. time VLD[+]		Fl. time VSD[+]		Gene expression KD/KO*
					Days	Nodes	Days	Nodes	Days	Nodes	
Medtr2g059540	*CDF1*[3]	*CDF1*	NF9016	exon 1	65.1±1.49	17.6±0.28	23.1±0.61	8.4±0.51	63.1±4.00	13.8±0.81	yes
Medtr5g041420	*CDFc2_1*	*CDF2*	NF13527	exon 2	62.4±0.75	17.1±0.61	25.9±0.77	9.1±0.36	63.1±1.42	13.3±0.57	yes
Medtr5g041530	*CDFc2_4*	*CDF3*	NF21008	exon 2	nd	nd	28.7±1.84	8.8±1.23	69.4±0.70	14.4±0.43	yes
			NF21606	exon 2	65.9±0.80	18.3±0.41	21.5±0.76	7.7±0.68	52.6±1.63	12.8±0.33	yes
Medtr6g012450	*CDFb2*	*CDF4*	NF0134	no Tnt1	na	na	na	na	na	na	na
			NF10121	no Tnt1	na	na	na	na	na	na	na
			NF11690	intron 1	61.8±0.87	16.8±0.36	24.4±1.17	8.6±0.44	nd	nd	no
Medtr6g027460	*CDFd1_1*[3]	*CDF5*	NF9161	no Tnt1	na	na	na	na	na	na	na
			NF9868	exon 2	nd	nd	19.0±1.15	6.0±0.00	nd	nd	yes
			NF20445	no Tnt1	na	na	na	na	na	na	na
Medtr7g010950	*CDFb1*	*CDF6*	NF6504	intron 1	nd	nd	20.5±6.35	7.0±0.00	nd	nd	no
			NF14323	intron 1	63.5±2.76	17.2±0.81	22.3±1.22	8.1±0.42	nd	nd	no
			NF13712	no Tnt1	na	na	na	na	na	na	na
Medtr2g016030	*CDFe*[3]	*CDFa*	NF7191	exon 1	58.7±2.63	16.4±0.54	28.3±1.59	9.9±0.43	69.5±0.77	14.4±0.63	yes
			NF16323	no Tnt1	na	na	na	na	na	na	na
Medtr3g435480	*CDFa2*	*CDFb*	NF9123	exon 2	66.1±2.14	18.2±0.56	27.9±0.83	10.5±0.34	68.0±0.99	13.6±0.46	yes
			NF11050	exon 2	nd	nd	30.2±2.50	11.1±0.83	nd	nd	yes
Medtr4g082060	*CDFc1*	*CDFc*	NF1963	no Tnt1	na	na	na	na	na	na	na
			NF14291	exon 2	55.3±2.42	17.2±0.67	23.6±1.30	8.5±0.70	65.5±0.78	14.0±0.40	yes
Medtr5g041380	*CDFc2_3*	*CDFd*	NF2921	no Tnt1	na	na	na	na	na	na	na
			NF4354	exon 1	64.1±2.31	16.4±0.80	27.7±0.89	10.4±0.39	59.5±0.98	12.9±0.38	yes
Medtr5g041400	*CDFc2_2*	*CDFe*	NF11400	exon 1	59.9±3.13	16.3±0.64	28.7±1.59	10.7±0.83	60.2±0.82	13.9±0.47	yes
			NF17470	exon 2	nd	nd	21.6±0.73	7.4±0.60	nd	nd	yes
Medtr6g027450	*CDFd1_2*[3]	*CDFf*	NF1515	exon 2	nd	nd	20.1±0.98	7.2±0.64	nd	nd	nd
			NF19175	exon 2	63.4±4.66	17.5±1.61	22.3±1.07	7.8±0.30	61.1±5.08	13.6±0.73	yes
Medtr7g086780	*CDFd1_3*[3]	*CDFg*	NF11642	exon 2	57.3±1.99	16.1±0.46	24.1±0.45	8.8±0.32	59.9±0.77	13.9±0.34	yes
			NF21417	exon 2	nd	nd	20.9±1.36	6.8±0.64	nd	nd	nd
Medtr8g044220	*CDFd2*[3]	*CDFh*	NF16568	no Tnt1	na	na	na	na	na	na	na
R108 WT					58.0±0.78[a]	15.5±0.34[a]	23.9±0.59[c]	8.5±0.30[c]	58.7±1.00[g]	13.0±0.36[g]	
					54.4±0.75[b]	16.0±0.26[b]	22.7±0.96[d]	7.6±0.48[d]	69.5±0.58[h]	13.4±0.40[h]	
							26.1±0.65[e]	8.6±0.40[e]	63.3±1.34[i]	12.9±0.42[i]	
							24.5±0.68[f]	8.2±0.42[f]			

Fl., flowering; WT, wild type; nd, not done; na, not applicable; KD/KO, knock-down/knock-out

[1] Medicago gene identifiers from JCVI Medicago genome Mt4.0, http://www.jcvi.org/medicago/

[2] Gene names based on nomenclature from Ridge et al. 2016; [3] Additional extended nomenclature based on clades seen in Supplementary Figure 1

[4] Nomenclature from Thomson et al. 2019

* Based on qRT-PCR and/or full length cDNA amplification

[+] Values expressed as means ± 95% confidence intervals

[a] Same batch as NF9016, NF13527, NF21606, NF11690 and NF14323 in LD; [b] same batch as NF7191, NF9123, NF14291, NF4354, NF11400, NF19175 and NF11642 in LD; [c] same batch as NF9016, NF13527, NF21008, NF21606, NF11690 and NF14323 in VLD; [d] same batch as NF9868 and NF6504; [e] same batch as NF7191, NF9123, NF11050, NF14291, NF4354, NF11400, NF17470 and NF11642 in VLD; [f] same batch as NF1515, NF19175 and NF21417 in VLD; [g] same batch as NF9016, NF13527, NF21008 and NF21606 in VSD; [h] same batch as NF7191, NF9123 and NF14291 in VSD; [i] same batch as NF4354, NF11400, NF19175 and NF11642 in VSD

Supplementary Table S2: Primers used in this study

Primer name	Primer sequence	Primer used for
GW_Medtr2g059540-F (MtCDF1)	GGGGACAAGTTTGTACAAAAAAGCAGGCTATGGCAGATGGAAATCTTTTCTG	Gateway cloning
GW_Medtr2g059540-R (MtCDF1)	GGGGACCACTTTGTACAAGAAAGCTGGGTTCATGGAAGATGAAAAAGAGAGGA	
GW_Medtr5g041420-F (MtCDFc2_1)	GGGGACAAGTTTGTACAAAAAAGCAGGCTATGATTAATAATTCAACCATAACGCTT	
GW_Medtr5g041420-R (MtCDFc2_1)	GGGGACCACTTTGTACAAGAAAGCTGGGTGCCTTCAAACCAAGGTGTC	
GW_Medtr5g041530-F (MtCDFc2_4)	GGGGACAAGTTTGTACAAAAAAGCAGGCTAGCAAAAACCTACCTGAGATGACT	
GW_Medtr5g041530-R (MtCDFc2_4)	GGGGACCACTTTGTACAAGAAAGCTGGGTACACCGGACACACCTTCAAA	
GW_Medtr6g012450-F (MtCDFb2)	GGGGACAAGTTTGTACAAAAAAGCAGGCTATGTCTGAAGCAAAAGACCCTG	
GW_Medtr6g012450-R (MtCDFb2)	GGGGACCACTTTGTACAAGAAAGCTGGGTTGCTGTGTCAATCGGCTCAT	
GW_Medtr6g027460-F (MtCDFd1_1)	GGGGACAAGTTTGTACAAAAAAGCAGGCTCACAAAAATGAATGGTGAAGGTGAC	
GW_Medtr6g027460-R (MtCDFd1_1)	GGGGACCACTTTGTACAAGAAAGCTGGGTACAGAATCTCATGTGTGTTTGCC	
GW_Medtr7g010950-F (MtCDFb1)	GGGGACAAGTTTGTACAAAAAAGCAGGCTCTGTTTTTAGCAACAAAAATGTTGGA	
GW_Medtr7g010950-R (MtCDFb1)	GGGGACCACTTTGTACAAGAAAGCTGGGTGGTTGAACACGGCCAAATCA	
GW_Medtr2g016030-F (MtCDFe)	GGGGACAAGTTTGTACAAAAAAGCAGGCTAATATGGCTGAAGTGAGAAAAGGTG	
GW_Medtr2g016030-R (MtCDFe)	GGGGACCACTTTGTACAAGAAAGCTGGGTACAAGAAATCACAAGGAGGATGCTA	
GW_Medtr3g435480-F (MtCDFa2)	GGGGACAAGTTTGTACAAAAAAGCAGGCTAAAAATGATTGAAACTAAGGACCCT	
GW_Medtr3g435480-R (MtCDFa2)	GGGGACCACTTTGTACAAGAAAGCTGGGTTGACTAACCTCAATTCTCAAACACA	
GW_Medtr4g082060-F (MtCDFc1)	GGGGACAAGTTTGTACAAAAAAGCAGGCTTGAGTTGCACACAGATTTTCCT	
GW_Medtr4g082060-R (MtCDFc1)	GGGGACCACTTTGTACAAGAAAGCTGGGTTGCAATTCACAACTAGGTTTCCT	
GW_Medtr6g027450-F (MtCDFc1_2)	GGGGACAAGTTTGTACAAAAAAGCAGGCTTGGTGTGTTTTTGAGCTGTT	
GW_Medtr6g027450-R (MtCDFc1_2)	GGGGACCACTTTGTACAAGAAAGCTGGGTATTACCGGAGCGACAACTAT	
GW_Medtr7g086780-F (MtCDFd1_3)	GGGGACAAGTTTGTACAAAAAAGCAGGCTATGTCTGAGACTCAGAAGGATC	
GW_Medtr7g086780-R (MtCDFd1_3)	GGGGACCACTTTGTACAAGAAAGCTGGGTTTATATGCTTTCCTGGATTGTATG	
Medtr2g059540- F (MtCDF1)	TGTGGTACAGCATGGAGACTC	qRT-PCR
Medtr2g059540-R (MtCDF1)	AGACTGATCAACGGTGTTAGCA	
Medtr5g041420-F (MtCDFc2_1)	ACCATCTTGGAGCGTGCAAT	
Medtr5g041420-R (MtCDFc2_1)	AGCTGCAGGGTTAGAAGCAA	
Medtr5g041530-F (MtCDFc2_4)	AAGCGCGTGTTGATCCCTAA	
Medtr5g041530-R (MtCDFc2_4)	CCATGAAAAGCTCTCCCCCA	
R108_Medtr6g027460-F (MtCDFd1_1)	ATGGTGGCCGTTACAAACAT	
R108_Medtr6g027460-R (MtCDFd1_1)	TGGACACATTTCCATTCCTG	
Medtr2g016030-F (MtCDFe)	TTCATTAGCATCCTCCTTGTGA	
Medtr2g016030-R (MtCDFe)	CACATGGGAGGAAAATGGTT	
Medtr3g435480-F (MtCDFa2)	TTGGTTTCCGCCTCATACTC	
Medtr3g435480-R (MtCDFa2)	CATTTTCGTCGTTGGTGTTG	

Medtr4g082060-F (MtCDFc1)	ATCCTCCACTTTGCAACACC	
Medtr4g082060-R (MtCDFc1)	TTTGAAGCCGTAACCGAAAC	
R108_Medtr5g041380-F (MtCDFc2_3)	ATCAAGAATGGTGGAGGACT	
R108_Medtr5g041380-R (MtCDFc2_3)	AGCACGCCTTCAACATATTC	
Medtr5g041400-F (MtCDFc2_2)	TTGGGAATATGTAGCACCGATA	
Medtr5g041400-R (MtCDFc2_2)	GGACGTTGAATCGACAGGAC	
Medtr6g027450-F (MtCDFc1_2)	GCCGAACATTGGCTTGCAAT	
Medtr6g027450-R (MtCDFc1_2)	TCTCTGGTGTGTTTTCCAAGG	
Medtr7g086780-F (MtCDFd1_3)	AGATTTTGGAAGCCAGCTCA	
Medtr7g086780-R (MtCDFd1_3)	AGAGAAACAAAGGCCAGCAA	
MtFTa1-F	GTAGCAGTAGGAATCCACTAGC	
MtFTa1-R	ACACTCACTCTCGGTTGATTTCC	
MtFTb1-F	ATGAACCCTCTTGTGGTCTG	
MtFTb1-R	TGGATTGACTATTTGGGAAG	
MtFTb2-F	ACAAATCCTCTTGTTGTTGG	
MtFTb2-R	TGAGTTGATTATTTGAGAGG	
MtFTa2-F	AAGTGGTAGCAGACCGAATC	
MtFTa2-R	CACCACCATTGGTAACAGTC	
MtSOC1a-F	GCGTTGTTCGAGCAAGAAAGAATCAGGC	
MtSOC1a-R	GGGGCTGCTTAGAGAGCCTGGCATTT	
MtCOLa-F	GGTTGTGAGGTGGAGGAAGA	
MtCOLa-R	CAACAAAAGCCATGAAGCTG	
MtCOLb-F	CCGAACACGAACATGAACAC	
MtCOLb-R	CATCCATCGACGGTGAAGA	
MtCOLc-F	ATCTTCCTCGATGGACGTTG	
MtCOLc-R	GTCCCGTAACCACAGTTTGA	
MtCOLd-F	GCCGATGATGAATCACAACTCAG	
MtCOLd-R	GTGAAGATGACGAAACGCTATG	
MtCOLh-F	GAATAGAGGGGATGCCATGTT	
MtCOLh-R	CAAATCGCCCTCTCACTCTC	
Medtr2g059540_genotyping-F (MtCDF1)	TCGCTACTGGACCAAAGGTG	Genotyping
Medtr2g059540_genotyping-R (MtCDF1)	ATCACCGAGTCTCCATGCTG	
Medtr5g041420_genotyping-F (MtCDFc2_1)	AACGCTTTTTGGGAGAACGA	
Medtr5g041420_genotyping-R (MtCDFc2_1)	TGGAACAACTTGGTCTGGTTTC	
Medtr5g041530_genotyping-F (MtCDFc2_4)	ACCCAAAATCTCCCTCGTCG	
Medtr5g041530_genotyping-R (MtCDFc2_4)	CCATGAAAAGCTCTCCCCCA	
Medtr6g012450_genotyping-F (MtCDFb2)	CCGTGTCCCCGTTGTTATAG	
Medtr6g012450_genotyping-R (MtCDFb2)	GGCGGTGGTTGTACTTGAGA	
Medtr6g012450_genotyping-R2 (MtCDFb2)	AATCGCATCTGCCTTATCGT	
Medtr6g027460_genotyping-F (MtCDFd1_1)	CAATGTCAATCAACCTCGGCA	

Medtr6g027460_genotyping-R (MtCDFd1_1)	TGGACACATTTCCATTCCTG	
Medtr6g027460-F (MtCDFd1_1)	GCTACCCTGCATCTTCATGGA	
Medtr6g027460-R (MtCDFd1_1)	GCATTGCAGTAGGATCAATGCC	
Medtr7g010950_genotyping-F (MtCDFb1)	TCGTGTTGGAGATGTTGTTGT	
Medtr7g010950_genotyping-F1 (MtCDFb1)	GGAGGTTCATGTTGGAGATG	
Medtr7g010950_genotyping-F2 (MtCDFb1)	AGTGAAGAACAAGGCGAGGA	
Medtr7g010950_genotyping-R (MtCDFb1)	CCGATGGTTTTTCTCCCAATGT	
Medtr7g010950_genotyping-R1 (MtCDFb1)	CGGAATCACGTTGAGTTTCA	
Medtr7g010950_genotyping-R2 (MtCDFb1)	TGAAGCGACCTAGACAATGC	
Medtr2g016030_genotyping-F (MtCDFe)	CGGAAAGTTGTCTCTACGAAGC	
Medtr2g016030_genotyping-R (MtCDFe)	TGAATAACCACCTGAGGTCTTCC	
Medtr3g435480_genotyping-F (MtCDFa2)	TGGTAGAGTTCTCAACTTTGGGT	
Medtr3g435480_genotyping-R (MtCDFa2)	ATTGCGCCAGAACTCCAAGT	
Medtr4g082060_genotyping-F (MtCDFc1)	ACTGATCATCCCTGCCAACG	
Medtr4g082060_genotyping-R (MtCDFc1)	ACGAGGCTGGTTGACATTGT	
Medtr5g041380_genotyping-F (MtCDFc2_3)	ACAACACCGATGTTCCTACCA	
Medtr5g041380_genotyping-R (MtCDFc2_3)	CCTCTGTGGTTTGTTTTGAGGC	
Medtr5g041400_genotyping-F (MtCDFc2_2)	ACACGCAACACCGACGATTA	
Medtr5g041400_genotyping-R (MtCDFc2_2)	GAGATTTTGTTCTTGCGGCG	
Medtr6g027450_genotyping-F (MtCDFc1_2)	CCCGACAAAATTGTTCAATGTCC	
Medtr6g027450_genotyping-R (MtCDFc1_2)	GGTTGAAATTCCACCGGAAGC	
Medtr7g086780_genotyping-F (MtCDFd1_3)	CGATGCAACAGTTGGGACAC	
Medtr7g086780_genotyping-R (MtCDFd1_3)	TCGAACCGGAACACACTTGT	
Medtr8g044220_genotyping-F (MtCDFd2)	GATGCAGTACCTGTCATACACAT	
Medtr8g044220_genotyping-R (MtCDFd2)	AGAAGAGCTGGAAGAGTTCTCA	
Tnt1-F	ACAGTGCTACCTCCTCTGGATG	
Tnt1-R	CAGTGAACGAGCAGAACCTGTG	

Supplementary References

Imaizumi, T., Schultz, T. F., Harmon, F. G., Ho, L. A., and Kay, S. A. (2005). FKF1 F-box protein mediates cyclic degradation of a repressor of *CONSTANS* in Arabidopsis. *Science* 309, 293–297. doi:10.1126/science.1110586.

Ridge, S., Sussmilch, F. C., Hecht, V., Schoor, K. Vander, Lee, R., Aubert, G., et al. (2016). Identification of *LATE BLOOMER2* as a *CYCLING DOF FACTOR* homolog reveals conserved and divergent features of the flowering response to photoperiod in pea. *The Plant Cell* 28, 2545–2559. doi:10.1105/tpc.15.01011.

Thomson, G., Taylor, J., and Putterill, J. (2019). The transcriptomic response to a short day to long day shift in leaves of the reference legume *Medicago truncatula*. *PeerJ* 7, e6626. doi:10.7717/peerj.6626.
